# Potential Role of Natural Antioxidants in Countering Reperfusion Injury in Acute Myocardial Infarction and Ischemic Stroke

**DOI:** 10.3390/antiox12091760

**Published:** 2023-09-13

**Authors:** Sofía Orellana-Urzúa, Camilo Briones-Valdivieso, Silvia Chichiarelli, Luciano Saso, Ramón Rodrigo

**Affiliations:** 1 Molecular and Clinical Pharmacology Program, Institute of Biomedical Sciences, Faculty of Medicine, University of Chile, Santiago 8380000, Chile; sofiorellana@ug.uchile.cl; 2 Facultad de Medicina, Universidad Diego Portales, Santiago 8370007, Chile; camilo.briones@mail.udp.cl; 3 Department of Biochemical Sciences “A. Rossi-Fanelli”, Sapienza University of Rome, 00185 Rome, Italy; silvia.chichiarelli@uniroma1.it; 4 Department of Physiology and Pharmacology “Vittorio Erspamer”, Faculty of Pharmacy and Medicine, Sapienza University, P.le Aldo Moro 5, 00185 Rome, Italy; luciano.saso@uniroma1.it

**Keywords:** oxidative stress, reperfusion injury, antioxidants, ischemic stroke, acute myocardial infarction

## Abstract

Stroke and acute myocardial infarction are leading causes of mortality worldwide. The latter accounts for approximately 9 million deaths annually. In turn, ischemic stroke is a significant contributor to adult physical disability globally. While reperfusion is crucial for tissue recovery, it can paradoxically exacerbate damage through oxidative stress (OS), inflammation, and cell death. Therefore, it is imperative to explore diverse approaches aimed at minimizing ischemia/reperfusion injury to enhance clinical outcomes. OS primarily arises from an excessive generation of reactive oxygen species (ROS) and/or decreased endogenous antioxidant potential. Natural antioxidant compounds can counteract the injury mechanisms linked to ROS. While promising preclinical results, based on monotherapies, account for protective effects against tissue injury by ROS, translating these models into human applications has yielded controversial evidence. However, since the wide spectrum of antioxidants having diverse chemical characteristics offers varied biological actions on cell signaling pathways, multitherapy has emerged as a valuable therapeutic resource. Moreover, the combination of antioxidants in multitherapy holds significant potential for synergistic effects. This study was designed with the aim of providing an updated overview of natural antioxidants suitable for preventing myocardial and cerebral ischemia/reperfusion injuries.

## 1. Introduction

Acute ischemic diseases, such as acute myocardial infarction (AMI) and ischemic stroke (IS), stand as major causes of death and disability worldwide [1]. Both conditions result from the occlusion of a vascular structure [2,3]. The acute management for them is the prompt restoration of blood flow to the tissue [4,5], thereby reducing the time of hypoperfusion to preserve organ function [6]. Paradoxically, the restoration of blood flow induces an important additional damage [7,8], which is a phenomenon known as ischemia–reperfusion injury (IRI). This phenomenon was first described by Jennings et al. [9], in relation to the myocardium in 1960, and by Ames et al. [10], in relation to the brain, in 1968. While cardiac ischemia/reperfusion is associated with necrosis, cardiomyocyte apoptosis, contractile dysfunction, and life-threatening ventricular arrhythmias, cerebral IRI induces an increase in trans-endothelial permeability and blood–brain barrier (BBB) damage [11]. While essential for restoring aerobic ATP production, the re-entry of oxygenated blood into ischemic tissue leads to elevated reactive oxygen species (ROS) production. This effect can induce oxidative modifications in nearly all types of biomolecules within cells, ultimately resulting in cell dysfunction. This phenomenon has been labeled the ‘oxygen paradox’ [12], wherein oxidative stress-mediated injury and ischemia reperfusion damage play pivotal roles. However, the pathophysiology of IRI involves a complex interplay of mechanisms, including oxidative stress (OS), endoplasmic reticulum stress, calcium overload, inflammatory response, disturbances in energy metabolism, apoptosis, and various forms of programmed cell death (e.g., necroptosis, autophagy, pyroptosis, patanatos, and ferroptosis) [1].

IRI also comprises other mechanisms, such as microvascular vasoconstriction, whereas endothelin in the vascular wall can increase in OS, contributing to the reduction in NO bioavailability and consequently leading to vascular dysfunction [13]. Also, IRI is characterized by tissue necrosis and leukocyte infiltration, especially neutrophils that are recruited to the ischemic site following reperfusion and, prior to extravasation, adhere to the endothelium. Interestingly, protection against IRI has been shown by blocking neutrophil–endothelial cell adhesion in vivo [14].

Hypoxia-inducible factor-1α (HIF-1α), an oxygen-sensitive transcription factor that mediates adaptive metabolic responses to hypoxia, is activated in IRI by improving mitochondrial function and decreasing OS, thus accounting for protective cellular mechanisms [15]. HIF-1α activates the transcription of other genes which play an important role in a cell’s adaptive responses to hypoxia, such as vascular endothelial growth factor (VEGF), erythropoietin (EPO) and glucose transporter-1. Among them, VEGF is the most important angiogenetic factor in all steps of angiogenesis [16]. During ischemia reperfusion, VEGF activity decreases due to the increase in its antagonist soluble fms-like tyrosine kinase-1 (sFlt-1), which is a truncated form of the VEGF receptor fms-like tyrosine kinase-1 (Flt-1) lacking the transmembrane and cytoplasmic domains. Therefore, increased sFlt-1 results in a harmful effect on the heart following MI, and it has been positively related to the severity and mortality of these patients [17].

Also, microRNAs (miRs) have been shown to be involved in the regulation and pathogenesis of ischemia [18] and IRI in the heart [19] as well as in the central nervous system [20]. They are tissue-specific [21] and are capable of silencing target genes, and thus, among other functions, miRs regulate OS by targeting ROS producers, ROS pathways and antioxidant effectors. Their functionality varies across contexts; some miRs exhibit pro-oxidant effects, while others concurrently target genes with opposite redox regulatory functions [22].

The cellular oxidative status is determined by the equilibrium between ROS formation and the activity of various antioxidant systems [23]. The mechanisms of damage are mainly mediated by ROS-induced transcription factors such as nuclear factor kappa B (NF-κB) and activator protein-1 (AP-1). During ischemia followed by reperfusion, ROS mediate NF-κB activation by phosphorylating the IκB (NF-κB inhibitor proteins family) subunit, thereby provoking its proteolytic digestion. Then, NF-κB is translocated into the nucleus, leading to the transcription of genes causing inflammation and apoptosis [24]. Also, ischemia reperfusion leads to c-Jun N-terminal kinase (JNK) phosphorylation, which is translocated into the nucleus, inducing the expression of inflammation and apoptosis-related genes and AP-1 activation. It was reported that AP-1-induced an upregulation of soluble epoxide hydrolase, which is implicated in IRI [25].

The antioxidant defense system (ADS) is regulated by transcriptions factors, such as nuclear factor erythroid 2-related factor 2 (Nrf2), which is a critical regulator of the cellular stress response. It controls cellular defense responses against OS by modulating the expression of antioxidant enzymes at the transcriptional, post-transcriptional and post-translational levels [26]. The ADS operates through both enzymatic and non-enzymatic molecules. Key enzymes include superoxide dismutases (SODs), catalase (CAT), peroxiredoxins, thioredoxins, glutaredoxins, and glutathione peroxidases (GPXs). Non-enzymatic molecules can be classified as endogenous, such as reduced glutathione, and exogenous, including vitamin C, vitamin E, carotenoids, flavonoids, and polyphenols, among others [24,27,28]. It is noteworthy that natural antioxidants constitute a diverse group of exogenous molecules that can be considered as pharmacological resources due to their safety, availability, and potential pleiotropic benefits against oxidative challenges.

Plenty of evidence suggests that OS plays a significant role in the pathogenesis of IRI [6], encompassing AMI and IS. Consequently, the objective of this review is to present an updated overview of the potential role of natural antioxidant molecules in preventing OS-induced damage during ischemia followed by reperfusion injury in the heart and brain. This aim suggests the hypothesis that combining these compounds should improve the benefits achieved by individual monotherapies.

## 2. Acute Myocardial Infarction

Myocardial reperfusion injury can result from four recognized forms of damage: reperfusion-induced arrhythmias, myocardial stunning, microvascular obstruction, and lethal myocardial reperfusion injury. The latter involves the death of cardiomyocytes that were viable at the end of the ischemic event and contributes up to 50% of the final myocardial infarct size [29]. While OS itself can induce injury by damaging macromolecules such as proteins, lipids, DNA, and enzymes [30], and by activating the NF-κB pathway [31], it is important to note that OS also enhances other injury mediators. After reperfusion, the combined impact of restoring physiological pH levels and calcium overload plays an important role in the opening of the mitochondrial permeability transition pore (mPTP) [32], which is a critical mechanism in myocardial IRI [33]. ROS further enhances this process both through direct modifications to the pore and indirectly by exacerbating intracellular calcium overload due to the damage it can cause in the sarcoplasmic reticulum [29] as well as in the type 2 ryanodine receptor [34,35,36].

**Figure 1:**
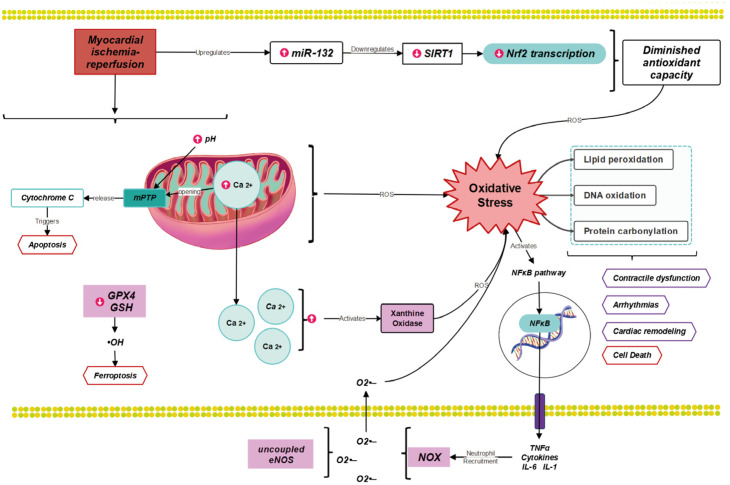
Role of oxidative stress in myocardial damage caused by ischemia followed by reperfusion. The increase in cytosolic calcium is associated with the activation of XO, which is an enzyme with pro-oxidant properties. The increase in calcium inside the mitochondria and the increase in pH during reperfusion lead to the opening of the mPTP, which causes the decrease in the mitochondrial transmembrane potential linked to ROS generation. These reactive oxygen molecules activate the transcription factor NF-κB, promoting inflammation and neutrophil migration to the injured region as well as increasing ROS production by NADPH oxidase. Also, ROS originates from uncoupled eNOS, oxidizing lipids, proteins, and DNA, triggering cell death. In addition, apoptosis is induced by the release of cytochrome c via mPTP, whereas ferroptosis is inducted by reduced GSH and GPX4 activity, leading to the accumulation of lipid peroxidation products. Furthermore, miR-132 directly targets SIRT1 and negatively regulates its expression, leading to a decrease in Nrf2 transcription, which in turn results in diminished antioxidant capacity; Ca^2+^: calcium; DNA: deoxy-ribonucleic acid; NF-κB: nuclear factor kappa B; eNOS: endothelial nitric oxide synthase; O2•−: superoxide anion; NOX: NADPH oxidases; TNFα: tumor necrosis factor-alpha; IL-6: interleukin 6; IL-1: interleukin 1; miR-132: microRNA-132; SIRT1: sirtuin 1; Nrf2: nuclear factor erythroid 2-related factor 2.

Early after myocardial reperfusion, there is a burst of ROS generation [37], including hydrogen peroxide (H_2_O_2_), superoxide radical anion (O_2_•−), hydroxyl radical (•OH), and peroxynitrite anion (ONOO−) [38]. In contrast, the most important ROS sources are NADPH oxidases (NOX), uncoupled endothelial nitric oxide synthase (eNOS), xanthine oxidase (XO) and the mitochondrion [31], the latter because of the oxidation of the ischemic accumulated succinate [39,40]. The mechanisms involved in IRI are summarized in Figure 1.

Non-coding RNAs also play a relevant role in this disease. During ischemia, miRs contained within cardiomyocytes are released into bloodstream, leading to cell damage [19]. This is important for considering them as serum biomarkers and predictors for some diseases. For example, in patients who have suffered an acute coronary syndrome, miR-1, miR-21, miR-29a, miR-30a, miR-34a, miR-146a, miR-150, and miR-208a expression have been associated with left ventricular remodeling, while the expression levels of miR-150 could serve as a positive predictor [18]. In a recent review, the diagnostic and therapeutic roles of cardiac miRs were studied [41]. Among the potential therapeutic targets in cardiac ischemia reperfusion, it was reported that the infarct size can be reduced by the inhibition of miR-15b [42], whereas an improvement of cardiac function and reduction in remodeling have been achieved by targeting miR-92a [43], miR-15b [42] and miR-208a-3p [44]. Also, miR-132 is of particular interest, because it is upregulated in myocardial IRI, and its inhibition could improve outcomes by inhibiting OS and pyroptosis, targeting sirtuin-1 (SIRT1) [18]. SIRT1 exerts antioxidative effects against myocardial IRI through strengthening the Nrf2 pathway [45]. On other hand, circular RNAs (circRNAs) play important roles in cardiac cell death after myocardial IRI. Ferroptosis-associated circRNA plays an important role in protecting against ischemia reperfusion-induced dysregulation of the redox state and excessive iron accumulation in a ferroptosis-dependent manner [46].

## 3. Cardiac Arrest

Cardiac arrest (CA) causes an interruption of blood flow to all tissues, resulting in a dysfunction of oxygen and metabolite availability. When blood flow and oxygen supply are resumed during resuscitative efforts, the process of reperfusion injury is initiated, which is characterized by the generation of ROS capable of causing direct tissue damage. Plasma plays a critical role by acting as a reservoir and transport medium for both oxygen and metabolites essential for survival as well as for ROS generation. A study examined the imbalance between prooxidant and antioxidant substances in plasma during the initial phase after resuscitation from CA. This imbalance leads to a predominance of prooxidants and is associated with increased levels of OS-induced end products, which the body’s antioxidant system cannot directly neutralize. Thus, circulating plasma plays a key role in the generation of OS after CA and underscores the need for early and substantial intervention to obtain positive results in its treatment [47]. Furthermore, a study conducted in 152 patients of out-of-hospital cardiopulmonary arrest showed that OS, indicated by biological antioxidant potential on admission, was strongly correlated with the neurological outcome after CA [48].

Some management alternatives have been proposed, such as hypothermia, which has been shown to attenuate the neurological deficits and hippocampal morphological changes induced by CA in rats. It has been hypothesized that the protective effect of hypothermia after CA may be related to the inhibition of OS and apoptosis, and its underlying mechanisms may be, at least in part, due to the activation of the glycogen synthase kinase 3 beta (GSK-3β)/Nrf2/heme oxygenase-1 (HO-1) pathway [49]. Similarly, a clinical study that included 31 patients under controlled normothermia and 11 patients treated with 24 h mild therapeutic hypothermia evidenced that hypothermia reduces malondialdehyde (MDA) and protein carbonyl levels, which are both markers of oxidative damage. Concomitantly, hypothermia increases the activity of antioxidant enzymes SOD, GPX, and glutathione S-transferase [50]. Thus, evidence shows that OS plays an important role in the outcomes following CA, and that patients suffering this pathology could benefit from antioxidant interventions to improve their prognosis.

## 4. Ischemic Stroke

Some aspects of ROS injury in the brain differ from those occurring in the heart. This distinction begins with the central nervous system’s particular vulnerability to ROS damage, which is possibly due to its high oxygen consumption [51] and relatively lower levels of antioxidant activity compared to the heart [12]. Also, ROS production occurs in three distinct phases; mitochondrial ROS generation occurs only during the first minutes of hypoxia, whereas after reperfusion, ROS are generated by XO activity and, mainly, by the calcium-dependent activation of NOX [52]. Neuronal injury caused by OS triggers microglia activation, an impairment of the BBB permeability, and the reactivation of peripheral immune responses [2]. Moreover, OS can cause neuronal death by several mechanisms, like necroptosis, a caspase-independent cell death mode, and ferroptosis, a form of regulated necrosis where there is excessive ROS caused by iron-dependent lipid peroxidation [53].

After an episode of ischemia, the brain’s endothelial cells and astrocytes produce a significant quantity of chemokines and cytokines. These substances then stimulate the expression of adhesion molecules on the endothelium, which in turn leads to the adhesion of leukocytes and the breakdown of tight junction proteins and the extracellular matrix within the endothelium. Ischemia followed by reperfusion causes a time-dependent recruitment and activation of inflammatory cells, including neutrophils, T cells, and monocytes/macrophages. Consequently, inhibiting such inflammatory responses can reduce the size of the infarction and mitigate neurological impairments. Importantly, the impaired BBB facilitates both the entry of peripheral inflammatory cells into the brain and the release of harmful mediators, resulting in sustained barrier damage [54]. miR-98 overexpression in activated endothelial cells decreased the secretion of pro-inflammatory factors, the expression of adhesion molecules, as well as the monocyte adhesion/migration across the BBB [54]. Also, circRNAs are associated with BBB damage: for example, circ-FoxO3 inhibited mechanistic target of rapamycin complex 1 (mTORC1) activity mainly by sequestering mTOR and E2F Transcription Factor 1, thus promoting autophagy to clear cytotoxic aggregates for improving BBB integrity [55].

**Figure 2:**
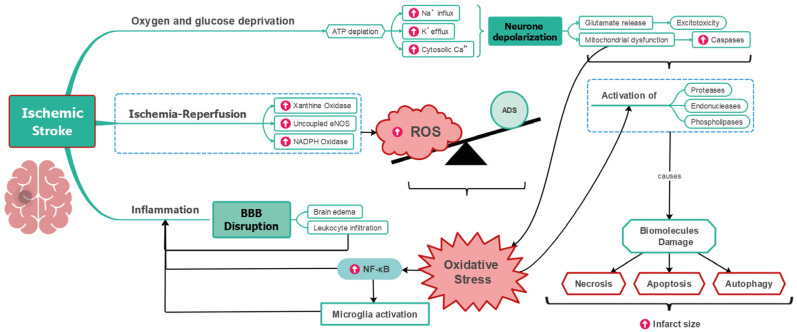
Role of oxidative stress in brain damage caused by ischemia followed by reperfusion. ATP: adenosine triphosphate; Na^+^: sodium; K^+^: potassium; Ca^2+^: calcium; ROS: reactive oxygen species; ADS: antioxidant defense system; BBB: blood–brain barrier; NF-κB: nuclear factor kappa B.

From a clinical perspective, it is crucial to acknowledge that early reperfusion is currently is the sole therapy employed to decrease the infarct size in patients with IS, where a significant proportion of them fail to achieve fully recovery [56]. This may be attributed in part to the lethal injury, caused by reperfusion, to neurons in the penumbra [57]. Thus, the development of new strategies for neuroprotection to improve clinical outcomes appears urgent [56]. Although many antioxidant agents are being investigated to prevent cerebral IRI, they are yet not commonly utilized in healthcare services, as their mechanisms of action and potential clinical consequences have not been precisely clarified [58]. The role of OS in stroke pathophysiology is summarized in Figure 2.

## 5. Natural Antioxidant Bioactive Molecules against Myocardial Infarction and Ischemic Stroke Injury

**Figure 3:**
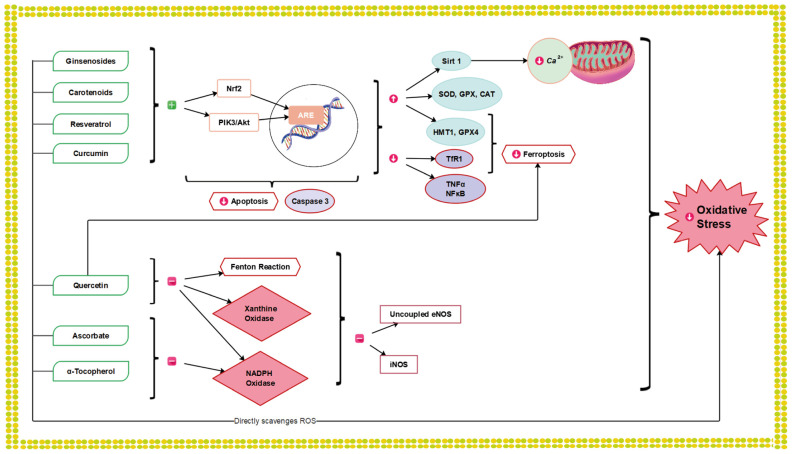
Antioxidant protective pathways involved in counteracting reperfusion injury. Nrf2: nuclear factor erythroid 2-related factor 2; PI3K: phosphoinositide 3-kinase; AKT: protein kinase B; ARE: antioxidant response elements; SIRT1: sirtuin 1; CAT: catalase; factor-alpha; NF-κB: nuclear factor kappaB; Ca^2+^: calcium; eNOS: endothelial nitric oxide synthase; iNOS: inducible nitric oxide synthase; NADPH oxidase: nicotinamide adenine dinucleotide phosphate oxidase; ROS: reactive oxygen species; SOD: superoxide dismutase; TNF-α: tumor necrosis factor alpha; GPX: glutathione peroxidase; HMT1: type 1 arginine methyltransferase; TfR1: transferrin receptor protein 1.

Natural antioxidants are present in low concentrations within cells. They reduce free radicals to provide a protection system against vascular diseases. They have a strong potential to inhibit OS, lipid peroxidation and the oxidation of breakdown products. Natural antioxidants can function either individually or synergistically to remove free radicals generated during oxidative metabolism, thus maintaining the redox balance [59]. In the following sections, the properties and potential roles of certain natural antioxidants that could serve as therapeutic pharmacological agents in AMI and IS will be presented. The antioxidant protective pathways engaged in counteracting reperfusion injury are summarized in Table 1 and Figure 3.

### 5.1. Phenolic Compounds

#### 5.1.1. Polyphenols

##### Resveratrol

Resveratrol, also known as 3,5,4′-trihydroxy-trans-stilbene, is a phytoalexin produced in various plants in response to injury caused by pathogens or physical damage. This compound can scavenge ROS and prevent lipid peroxidation in various OS related diseases. Foods such as grape skins, blackberries, raspberries, blueberries and peanuts contain resveratrol, being also one of the main components of red wine [92].

Resveratrol exists in either cis- or trans-resveratrol isoforms, the trans-isomer being more active as an antioxidant than the diastereomeric mixture [93]. This compound is also characterized by a good absorption capacity and rapid metabolization in the body, mainly through the formation of sulfoxide and glucuronide conjugates, which are mainly eliminated in the urine. In general, resveratrol has been found to be well tolerated, and no significant toxic effects associated with its consumption have been reported [94].

Resveratrol has been the subject of numerous preclinical and clinical studies due to its beneficial effects on various diseases, demonstrating its ability to reduce both OS and inflammation in different OS-dependent pathologies, both in animal models and in humans. Regarding the pathologies addressed in this article, resveratrol has been shown to have neuroprotective effects in both animal models and clinical studies of IS. This happens through a variety of mechanisms, mainly because of its antioxidant and anti-inflammatory capacities [95], among other mechanisms, some of them not fully elucidated. Resveratrol has been shown to promote neurogenesis while reducing neurotoxicity by modulating glial activity and signaling [96]. In addition, resveratrol may play a neuroprotective role by maintaining mitochondrial function, promoting neurogenesis and angiogenesis, thus contributing to maintaining the integrity and health of nerve cells [61]. Moreover, resveratrol exhibits significant SOD enzyme activation capacity, which has been shown to have an antioxidant effect in brain IRI [97,98]. Complementarily, resveratrol has also been shown to downregulate MDA levels in brain IRI [99].

To understand its protective effect in brain IRI, it is relevant to consider the Nrf2 and antioxidant response elements (ARE) signaling pathway. Normally, Nrf2 is found interacting with Kelch-like ECH-associated protein 1 (Keap1), forming the Keap1–Nrf2 complex, which limits Nrf2-mediated gene expression [100] However, when the Nrf2/ARE signaling pathway is activated, the Keap1–Nrf2 complex disintegrates, allowing Nrf2 to translocate to the cell nucleus, where it binds to ARE to activate the expression of antioxidant enzymes such as HO-1 and SOD. These enzymes, in turn, work to attenuate OS in cells [100].

This is precisely where resveratrol plays an important role, as it has been shown to ameliorate brain injury caused by OS due to brain ischemia reperfusion by regulating the expression of Nrf2 and HO-1. By activating the Nrf2/ARE pathway, resveratrol promotes intracellular antioxidant defense, reducing levels of OS and providing protection against brain damage in the context of ischemia reperfusion [101].

There is a variety of preclinical and clinical evidence regarding the beneficial effect of resveratrol following IS. In a study conducted in rodents, resveratrol demonstrated a remarkable ability to significantly decrease neurological deficit scores, reduce brain infarct size, mitigate neuronal injury and myeloperoxidase activity [102]. In the context of cerebral ischemia, increased expression levels of toll-like receptor 4 (TLR4), NF-κB p65, cyclooxygenase-2 (COX-2), matrix metalloproteinase-9 (MMP-9), tumor necrosis factor alpha (TNF-α) and interleukin (IL)-1 beta (IL-1β) were observed, but resveratrol was able to attenuate the activity of all these factors [102]. Thus, resveratrol exerts a beneficial effect by reducing inflammation, preserving the integrity of the BBB and protecting against brain damage in rats subjected to focal cerebral ischemia. Furthermore, these neuroprotective effects of resveratrol may be linked to its ability to downregulate the TLR4 pathway [102].

Interestingly, a study has highlighted the role of resveratrol preconditioning in providing a long-term window of tolerance to cerebral ischemia, extending up to 2 weeks in mice [103]. This preconditioning process has been shown to have a significant impact on bioenergetic efficiency through its effect on cellular pathways such as enhanced glycolysis, mitochondrial respiration efficiency, and increased energy production (increased tricarboxylic acid cycle) as well as regulated oxidative phosphorylation and pyruvate uptake [103]. Furthermore, a recent meta-analysis including 54 studies of rodent animal models of IS showed a significant decrease in infarct volume and improved neurobehavioral score in resveratrol sub-groups with a dosage of 20–50 mg/kg. Thus, resveratrol treatment presented neuroprotective effects in IS models [104]. Similarly, another recently published meta-analysis including a total of 41 studies in rats showed that compared to the control group, resveratrol significantly reduced brain infarct volume and brain water content after brain IRI, subsequently improving neurological function. In this study, the level of MDA decreased significantly after resveratrol treatment. The optimal therapeutic dose according to this study would be 30 mg/kg [61].

In summary, resveratrol shows significant antioxidant action by activating SOD and downregulating MDA in brain IRI. Furthermore, its ability to regulate the Nrf2/ARE signaling pathway and increase the expression of antioxidant enzymes such as HO-1 and SOD translates into a neuroprotective effect that helps mitigate the damage caused by OS in the brain subjected to ischemia and subsequent reperfusion. These findings highlight the therapeutic potential of resveratrol in the treatment of OS and ischemia reperfusion-related brain conditions.

Regarding cardiac protection, resveratrol acts directly and indirectly on molecular pathways [105]. Decades ago, resveratrol was shown to be able to suppress low-density lipoprotein (LDL) oxidation in humans [106] as well as to reduce lipid peroxidation [107]. Since then, several studies have expanded the evidence supporting the potential of resveratrol to combat cardiovascular diseases, mainly by acting on OS and inflammation.

In relation to its involvement in OS, resveratrol plays a crucial role as a component of the ADS not only by acting as a free radical scavenger but also by increasing the activity of antioxidant enzymes and regulating genes related to redox balance, nitric oxide (NO) availability and mitochondrial functionality [108]. The stimulation of endothelial NO production is achieved by the upregulation of eNOS expression [109]. In addition, resveratrol can reduce OS by inhibiting NOX. It also can inhibit vascular inflammation and prevent platelet aggregation.

Furthermore, several studies have shown that resveratrol exerts certain effects through modifications in sphingolipids, a category of biological lipids with multiple cellular functions, including apoptosis, cell proliferation, OS, and inflammation. These lipids have attracted considerable interest as critical emerging determinants in the risk and development of cardiometabolic diseases. The resveratrol-mediated modulation of sphingolipid metabolism and signaling may represent an essential mechanism by which the compound exerts its effects, including the maintenance of oxidative balance [110].

Given the importance of resveratrol in cardiovascular protection, a number of both preclinical and clinical studies have been conducted. A recent meta-analysis compiled evidence from small animal studies on the effect of resveratrol on myocardial ischemia/reperfusion injury. This study concluded that pretreatment with resveratrol significantly reduced infarct size after myocardial IRI, regardless of the duration of reperfusion, route of administration, or temporal regimen of pretreatment [60].

In addition, the effect of resveratrol has been tested in other models of myocardial injury such as that induced by chronic intermittent hypoxia. In a study in 34 rats, resveratrol was shown to protect against chronic intermittent hypoxia-induced myocardial injury by inhibiting OS and endoplasmic reticulum stress by upregulating the expression of antioxidant molecules through Nrf2 [111]. Another study with isolated rat hearts showed that resveratrol significantly improved the mechanical performance of the heart after myocardial ischemia and reperfusion, along with an improvement in the redox status of the heart, indicated by lower levels of MDA and an increase in CAT, SOD and GPX activities [112]. In line with these results, resveratrol was shown to protect against isoproterenol-induced myocardial infarction in rats, showing lower O_2_•− and MDA production, which was accompanied by an increase in SOD in resveratrol-treated rats [113].

##### Quercetin

Quercetin is a flavonoid that is widely found in commonly consumed foods such as fruit, vegetables, tea, and red wine. It is safe for human consumption, even in high doses [114,115]. This compound has a number of biological properties, including antioxidant, anti-inflammatory, anti-aggregating and anti-aging [116].

Quercetin has been extensively investigated for its effect on ameliorating OS. This compound has been found to have the ability to scavenge free radicals [117] and to induce the expression of the antioxidant enzyme HO-1, which in turn helps to reduce OS caused by the enzyme NOX [118]. In addition, it has been observed that quercetin can suppress the expression of NOX2, which is an isoform of the NOX system that plays an important role in the production of ROS, such as H_2_O_2_ [119]. The overproduction of NOX has been shown to contribute to neurotoxicity and cerebrovascular disease, and it has been implicated as a major source of ROS in the brain [120]. Another promising effect of quercetin is its ability to attenuate the expression and activity of XO, which is another relevant enzyme in ROS production [121]. In addition to its antioxidant effects, quercetin protects against apoptosis and ROS generation by preserving mitochondrial function and preventing the release of cytochrome c [122]. It has also been shown to inhibit the function of myeloperoxidase, which contributes to atherosclerosis by generating hypochlorous acid from H_2_O_2_ [123].

Regarding evidence in IS, a recent systematic review and meta-analysis aimed at evaluating the efficacy and possible mechanisms of quercetin in the treatment of focal cerebral ischemia showed that compared to the control group, the quercetin-administered groups exhibited a marked improvement in neurological function score and a significant effect on reducing infarct volume. In addition, it was shown that quercetin could alleviate BBB permeability and brain water content [123]. The mechanisms involved in quercetin’s action against focal cerebral ischemia are diverse and involve antioxidation, anti-apoptosis, anti-inflammation and a reduction in calcium overload [123].

Another study showed that quercetin administration after IS is able to improve behavioral function, possibly through the upregulation of melanocortin-4 receptor in the brain [124]. Similarly, a study performed in rats subjected to transient middle cerebral artery occlusion recently showed that quercetin attenuates IRI; the results showed that quercetin significantly reduced cerebral infarct volume, neurological deficit, BBB permeability and ROS generation via the SIRT1/Nrf2/HO-1 signaling pathway [125].

One of the limitations of using quercetin in patients undergoing IS is that its effectiveness varies substantially depending on the type of source plant, the dose and the chemical properties after processing [126]. In humans, quercetin has low bioavailability, and it is noteworthy that it poorly crosses the BBB [127]. For this reason, studies have been conducted that aim to increase the bioavailability of quercetin in the brain, such as the use of nano-encapsulation and enzymatic modification, among other strategies. Some of these strategies, such as nanoparticle encapsulation, achieve a 50-fold increase in the bioavailability of this antioxidant [128].

This compound has also been shown to reverse myocardial remodeling after myocardial ischemia. Studies in animal models have shown that quercetin can reduce ischemic–reperfusion injury in the heart and improve myocardial function. It has also been shown to inhibit myocardial fibrosis, increase mitochondrial energy metabolism, reduce inflammatory response and attenuate OS in the heart [129,130].

A double-blind, placebo-controlled, randomized clinical trial showed that quercetin supplementation (500 mg/day) in post-AMI patients for 8 weeks significantly elevated total antioxidant capacity and improved the quality of life in post-myocardial infarction patients with no effect in blood pressure [131].

In conclusion, quercetin shows impressive antioxidant action and offers therapeutic potential in protecting the heart against OS and ischemia reperfusion. Its multiple biological properties make it a molecule of great interest for future research and applications in the field of medicine and cardiovascular health.

##### Curcumin

Turmeric species, in particular *Curcuma longa L.*, have been extensively studied and found to possess a wide range of pharmacological properties, including anti-inflammatory, anti-diabetic, anti-cancer, antiproliferative, antithrombotic, antioxidant, hypotensive, hypocholesterolemic, antirheumatic and antiviral effects, among many others [132,133]. The active compound in turmeric extract is curcumin, which is a lipophilic polyphenol with proven safety and good tolerance at high oral doses [134].

Curcumin has demonstrated synergistic therapeutic effects, enhancing the efficacy of other drugs and compounds, such as antibiotics, anti-inflammatories and polyphenols. Regarding myocardial IRI, curcumin has been shown to protect white matter after IS by inhibiting microglia/macrophage pyroptosis through the suppression of NF-κB and inhibition of the nucleotide-binding domain and leucine-rich repeat containing family pyrin domain containing 3 (NLRP3) inflammasome, thus reducing injury and improving functional outcomes in mice [135].

Moreover, a recent study in rats subjected to middle cerebral artery occlusion/reperfusion evidenced that curcumin pretreatment ameliorated IS injury by protecting BBB integrity and synaptic remodeling, as well as inhibiting inflammatory responses, which resulted in improved neurological scores and reduced infarct size, by increasing the protein expression level of tight junction proteins zonula occludens-1 (ZO-1), occludin and claudin-5 in ischemic rat brains. In addition, pre-treatment with curcumin before stroke was shown to reduce the phosphorylation of NF-κB and MMP-9, which are central mediators of inflammation [136].

Furthermore, curcumin has been reported to decrease the expression of light chain 3 phosphatidylethanolamine conjugate (LC3-II) and HIF-1α while increasing P62 levels in an in vitro model of IRI [137]. LC3-II is a widely used marker to assess autophagy, which is a cellular process that plays a crucial role in the degradation and recycling of damaged or unneeded cellular components. On the other hand, HIF-1α is a transcription factor that is activated under conditions of hypoxia (low oxygen availability) and plays a role in the cellular response to oxygen deprivation. The reduced expression of LC3-II and HIF-1α, together with increased P62, results in decreased cell death and apoptosis. This suggests that curcumin may exert a protective effect by modulating the cellular response to IRI, regulating autophagy and adaptation to hypoxia [137].

Similarly, a study performed in mice showed that curcumin exerts neuroprotective effects by mitigating autophagic activities through mediation of the phosphoinositide 3-kinase (PI3K)/protein kinase B (Akt)/mTOR pathway while suppressing an inflammatory reaction by regulating the TLR4/p38/mitogen-activated protein kinase (MAPK) pathway [138].

Another trial showed that curcumin promotes neuronal survival in vivo and in vitro by exerting neuroprotective effects against ischemia injury and also inhibiting ischemia-induced mitochondrial apoptosis by restricting B-cell lymphoma 2 (Bcl)-2-associated X protein (Bax) activation, which may be one of the possible mechanisms underlying curcumin’s neuroprotective effects [139].

A study performed in rodents showed that tetrahydrocurcumin has neuroprotective properties by reducing brain edema, infarct size and neuronal leakage after focal cerebral ischemia. Tetrahydrocurcumin also shows antioxidant effects by reducing oxidative damage and enhances cytochrome c homocysteinylation through the activation of MMP-9 [140].

Curcumin also contributes to the protection of the cardiomyocytes through several mechanisms. It improves cardiac function after IRI by inhibiting extracellular matrix degradation and collagen synthesis through the transforming growth factor beta (TGFβ)/ mothers against decapentaplegic (Smad) signaling pathway. In addition, curcumin mitigates oxidative damage and reduces cardiomyocyte apoptosis through activation of the janus kinase (JAK) 2/signal transducers and activator of transcription (STAT) 3 pathway, thereby alleviating myocardial IRI.

Meta-analyses of both animal and clinical studies have demonstrated the positive effects of curcumin in improving myocardial infarct size, cardiac function and indices of myocardial injury, oxidation, apoptosis and inflammation in models of IRI [141]. Clinical studies have shown that curcumin can reduce the incidence of cardiac dysfunction and in-hospital myocardial infarction, which is probably due to its anti-inflammatory and antioxidant properties. The optimal dose of curcumin in animal studies appears to be around 200 mg/kg/day [141].

Curcumin’s antioxidant action is complemented by its ability to act as a natural chelating agent and induce enzymatic antioxidant response via the Keap1/Nrf2/ARE pathway [142]. It can also bind directly to pro-inflammatory molecules, such as TNF-α and COX-1/COX-2, exerting its anti-inflammatory effects. Its interaction with transporter proteins improves their solubility and bioavailability while it can alter the activity of enzymatic molecules and the biological properties of other proteins [143].

In conclusion, the pleiotropic effects of curcumin are derived from its ability to trigger numerous protective mechanisms, making it a promising therapeutic bioactive antioxidant compound. Although the molecular mechanisms underlying its cardioprotective effects have yet to be fully elucidated, it is likely that the maintenance of redox balance is crucial to its therapeutic efficacy. Further research is needed to fully understand its potential in the treatment of myocardial IRI.

#### 5.1.2. Phenolic Acids

Phenolic acids are compounds that have a carboxylic acid group and are present in a wide variety of foods of plant origin, such as the skin of fruits, grape seeds, tea, honey, peach, red wine, and the leaves of vegetables. They are usually found as amides, esters, or glycosides, and they are rarely in free form [144]. Phenolic acids are mainly divided into two subgroups: hydroxybenzoic acid and hydroxycinnamic acid [145]. These compounds are of interest since they possess considerable antioxidant activity.

An in vivo study shows that treatment with *Macrotyloma uniflorum* seed extract, which is rich in phenolic acids such as p-coumaric acid and ferulic acid, exerts a significant cardioprotective effect in rats exposed to isoproterenol, probably due to the potent antioxidant activity of phenolic acids, which protect the myocardium from the harmful effects of isoproterenol [146].

A study in rodents evidenced that pretreatment with syringic acid, an abundant phenolic acid widely present in different plants such as grapes, olives, dates and spices, is able to mitigate myocardial IRI by inhibiting mitochondria-induced apoptosis via the PI3K/Akt/GSK-3β signaling pathway [147]. Similarly, *Melissa officinalis L.* extract, rich in phenolic acids, has shown the ability to suppress OS in rat hearts, preserving cardiac architecture and preventing fibrosis [148].

Regarding the effects of phenolic acids on the brain, a study in rats subjected to cerebral IRI showed that compared with the control group, treatment with total phenolic acids derived from *Sargentodoxa cuneata* was able to decrease the neurological deficit score, as well as ameliorate focal cerebral IRI in rats, by decreasing tissue inflammation and apoptosis pathways, increasing nutrition factor to protect neurons, activating brain cell self-protection and improving histopathological changes in hippocampus and cortical areas of the brain [149].

### 5.2. Carotenoids

Carotenoids are lipophilic compounds that are found in a wide range of fruits, vegetables, and other natural sources. They serve important functions in plants, protecting chlorophyll and mitochondria, while in animals, they act as potent antioxidants and support various aspects of health [150,151]. Once consumed, carotenoids are absorbed in the intestines and transported to various tissues in the body through lipoproteins. One of the best-known carotenoids is beta-carotene, which is abundant in carrots, sweet potatoes, and green leafy vegetables. Beta-carotene is a precursor of vitamin A, which is a nutrient essential for vision, immune function and cell growth [151].

Another important carotenoid is lycopene, which is found mainly in tomatoes and tomato products. Lycopene is a powerful antioxidant and has been associated with a reduced risk of various types of cancer and some other diseases [152,153].

In humans, carotenoids play a crucial role as antioxidants, helping to neutralize harmful free radicals and protecting cells from oxidative damage. The consumption of carotenoid-rich foods has been associated with various health benefits, including a reduced risk of chronic diseases, such as cardiovascular disease, certain cancers, and age-related macular degeneration [152]. In conjunction with their antioxidant function, carotenoids have also been studied for their potential anti-inflammatory and immunomodulatory effects [152].

Several studies, whether in vitro, in vivo or clinical, have been conducted to evaluate the effect of carotenoids in both the prevention and treatment of IS. In 2004, a prospective case-control study with a 13-year follow-up showed that higher plasma levels of carotenoids, as markers of fruit and vegetable intake, are inversely related to the risk of IS [154]. Subsequently, multiple sources of evidence have confirmed these findings: a 2017 systematic review indicated that high dietary intakes of six major carotenoids (beta-carotene, lycopene, zeaxanthin, lutein, and astaxanthin) were associated with a lower risk of stroke and other cardiovascular outcomes [155].

Carotenoids present in natural plant products have been shown to provide neuroprotection through a variety of mechanisms. Some of the main mechanisms by which carotenoids exert their neuroprotective effect include the following.

1. Inhibition of neuroinflammation: Carotenoids have shown the ability to reduce inflammation in the central nervous system by inhibiting the production of pro-inflammatory cytokines and inflammatory cell activation.
2. Regulation of microglial activation: Carotenoids can modulate the activity of microglial cells, which are immune cells in the central nervous system, helping to limit excessive inflammatory response.
3. Protection against the excitotoxic pathway: Carotenoids may act as neuroprotective agents by preventing neuronal damage caused by excitotoxicity, which is a process that occurs when there is excessive overstimulation of glutamate receptors on neurons.
4. Modulation of autophagy: Carotenoids can influence autophagy, which is a cellular process that plays a crucial role in the removal of damaged cellular components, thus helping to maintain the integrity of neurons.
5. Reducing oxidative damage: Carotenoids are known for their potent antioxidant activity, enabling them to neutralize ROS and reduce cell damage caused by OS.
6. Activation of defensive antioxidant enzymes: Carotenoids can also stimulate the activity of endogenous antioxidant enzymes in the brain, thereby enhancing the nervous system’s ability to counteract the effects of OS.

These various mechanisms of action work together to protect and preserve neuronal health, suggesting that carotenoids could be a promising option for the development of therapeutic or preventive strategies in neurodegenerative diseases and other neurological disorders. However, there is controversial evidence regarding the benefits of carotenoid supplementation [156]. Thus, it is important to note that more research is needed to fully understand the effects and benefits of carotenoids in neuroprotection and their potential clinical application.

Regarding cardiac disease, several attempts have been made to evaluate the cardioprotective potential of antioxidants in natural plant products against myocardial injury due to reperfusion. In the late 1990s, a large multicenter case-control study was conducted in 10 European countries to investigate the protective effect of carotenoid and tocopherol concentrations in adipose tissue in relation to the occurrence of AMI. The results showed that lycopene remained an independent protective agent with an odds ratio of 0.52 for the 10th and 90th percentile contrast [157].

Thereafter, in vivo studies in rats also confirmed the protective effect of lycopene against myocardial reperfusion injury. In 2006, it was observed that the oral administration of lycopene for 30 days after surgically induced myocardial infarction and significantly prevented myocardial damage according to histopathological examinations, suggesting that beneficial lycopene possibly suppresses OS and thereby reduces myocardial injury [158]. Similarly, in 2012, a study that assessed the effect of lycopene on reperfusion injury following isoproterenol-induced myocardial infarction revealed that 30-day pretreatment with oral lycopene effectively prevented isoproterenol-induced alterations in hemodynamics, electrocardiogram, apoptotic changes and biochemistry [159].

Furthermore, another study in which the effects of 2-methoxycinnamaldehyde (2-MA) from *Cinnamomum cassia* were studied in rats concluded that its administration significantly decreases OS after surgically-induced myocardial infarction, increasing functional recovery and reducing myocardial injury by inducting HO-1 and its anti-inflammatory effects [160].

In 2016, an evaluation of intravenous lycopene use in surgically induced myocardial infarction was conducted, finding that lycopene supplementation by intravenous injection significantly reduced myocardial infarction during in vivo reperfusion in rodents as well as significantly inhibited fatty acid oxidation and the activation of JNK signaling during reperfusion [161].

Additionally, the protective role of crocin (present in *Crocus sativus*) in reperfusion injury after AMI has been investigated. Crocin was found to exert protective effects through the Akt/eNOS/GSK-3β axis not only toward isoproterenol-induced cardiotoxicity but also counteracting norepinephrine-induced myocardial hypertrophy [162]. Evidence shows that this extract has a cardioprotective effect by upregulating Nrf2 expression [162].

The role of oleanolic acid (derived from terpenes) as a cardioprotective agent against reperfusion injury after myocardial infarction in isolated rat hearts has also been investigated. Pretreatment with oleanolic acid was found to enhance the mitochondrial antioxidant mechanism through increased reduced glutathione and alpha-tocopherol, thus providing a cardioprotective effect [163].

Another carotenoid of interest is astaxanthin, which is a xanthophyll with proven antioxidant activity [164]. A meta-analysis of randomized controlled trials including 380 participants showed that compared with placebo, astaxanthin significantly reduced blood MDA concentration. This result was particularly significant in patients with type 2 diabetes mellitus [164].

Regarding the mechanism of action, in vivo studies support that the protective effects of astaxanthin on the myocardium work through the Keap1–Nrf2 signaling pathway and mitochondria-mediated apoptosis [165]. Similarly, another in vivo study showed that this compound suppresses OS via activating the Nrf2/HO-1 pathway, thereby ameliorating cardiomyocyte apoptosis and cardiac dysfunction in rats [166]. Its mechanism of action also appears to be linked to inhibition of the TLR4/NF-κB signaling pathway, thus suppressing the release of inflammatory cytokines, which can cause myocardial cell death [167].

In addition, there are studies that show that miRs could also be involved in the action of this compound. A study conducted in an in vitro model identified differentially expressed miRs and related target genes that, with astaxanthin pretreatment, protect cardiomyocytes from IRI [74]. Similarly, another in vitro study shows that this carotenoid is capable of exert a protective function in myocardial cells via regulating the expression of miR-138/HIF-1α axis [168].

Regarding its effect on the brain, a study in rodents showed that pretreatment with astaxanthin attenuated brain injury due to IRI, which may be closely related to the decrease in OS [75]. In addition, another study in vivo showed that astaxanthin significantly protects the brain from OS damage and reduces neuronal deficits due to IRI [169]. The beneficial effects on the brain appear to be dose-dependent, with the medium dose (45 mg/kg) of astaxanthin appearing to be most effective in reducing complications of ischemia in rodents [170].

Regarding the pathways involved in the protective effect of astaxanthin in cerebral infarction, a study evidenced that pretreatment with astaxanthin resulted in an increased protein expression of Nrf2 (nuclear), HO-1, Bcl-2, CAT, SOD, and GPX while decreasing the content of TNF-α, IL-1β, IL-6, MDA, Bax and Nrf2 (cytosolic) [171]. Therefore, astaxanthin is an interesting compound for potential use as a therapy to ameliorate ischemia reperfusion damage in both cardiovascular pathologies and stroke.

These investigations provide promising evidence for the cytoprotective potential of natural antioxidants in the context of brain and myocardial reperfusion injury. However, further studies are required to consolidate and deepen these findings as well as to fully understand the underlying mechanisms involved in their potential therapeutical action.

### 5.3. Vitamins

#### 5.3.1. Vitamin C

Ascorbic acid, also recognized under the epithet of vitamin C, represents a fundamental pleiotropic antioxidant that performs various functions in multiple cellular compartments, acting on water-soluble elements [172]. The mechanisms whereby vitamin C exerts its beneficial influences have been the subject of much research, and part of this process is based on its ability to directly decrease ROS [173]. In addition to its ability to behave as an ROS scavenger, ascorbic acid intricately downregulates several enzymes related to ROS production, endothelial dysfunction, platelet coagulation, and smooth muscle cell tension.

The major mechanisms by which ascorbate is able to modulate endothelial function include the upregulation of antioxidant enzymes, eNOS and phospholipase A2, together with a direct reduction in the activity of NOX, which is the major source of O_2_•− in the cardiovascular system [174,175]. Although the underlying rationale for these effects has not been completely unraveled, it has been reported that ascorbate may be involved in the regulation of NOX synthesis [176] as well as in its regulation at both the transcriptional and post-transcriptional levels [176,177]. Additionally, ascorbate has been found to enhance eNOS activity by preventing oxidation of the cofactor tetrahydrobiopterin, thereby avoiding the enzyme uncoupling. Thus, ascorbate, together with glutathione, forms a primary barrier against ROS [27,28,178].

One other aspect of relevance that deserves to be highlighted in relation to the ascorbate effect consists in the fact that in addition to its direct interventions in aqueous environments, this compound is capable of regenerating α-tocopherol in cell membranes by means of achieving the reduction in the α-tocopheroxyl radical to its original α-tocopherol form [179]. In this regard, ascorbate has been found to carry out the regeneration of α-tocopherol on both sides of lipid bilayers [180] and erythrocytes [181].

A study in rats subjected to transient middle cerebral artery occlusion showed that the parenteral administration of vitamin C significantly improved neurological deficits and reduced cerebral infarction and cerebral edema by attenuating both oxidative and nitrosative stress, inflammatory responses, and the resultant disruptions of BBB barrier and cerebral neuronal apoptosis [182].

In relation to the clinical evaluation of the efficacy of ascorbate in cardiovascular conditions, several studies have been performed in recent decades that have yielded remarkable results. For example, in a cross-sectional analysis of 2383 individuals, low serum vitamin C levels were found to be associated with inflammation and the severity of peripheral arterial disease in smokers [183]. Similar findings emerged in patients with hypertension [184]. Other studies illustrate that vitamin C administration improves endothelial function in various patient groups, such as those affected by coronary artery disease, hypercholesterolemia, and cardiac allograft vasculopathy.

A meta-analysis involving a total of fifteen prospective cohort investigations and three prospective evaluations within intervention studies (with 320,548 participants and 16,974 cases) demonstrated that higher vitamin C intake, as well as higher circulating concentrations of vitamin C, vitamin E, and β-carotene, were associated with a reduced risk of cardiovascular disease mortality [185].

In a clinical trial, 56 patients were enrolled in a prospective, single-center, randomized study in which the infusion of 1 g of vitamin C (administered at a rate of 16.6 mg/min for 1 h prior to percutaneous coronary intervention) was compared against placebo. Patients undergoing percutaneous coronary intervention, after treatment exhibited a smaller decrease in myocardial perfusion compared to the placebo group. With statistically significant differences, complete microcirculatory reperfusion (with a thrombolysis in myocardial infarction (TIMI) perfusion grade = 3) was achieved in 79% of the vitamin C-treated group in contrast to 39% of the placebo group. These findings hint that in patients undergoing scheduled coronary angioplasty, an improvement of microcirculatory reperfusion is benefited by vitamin C infusion, suggesting an involvement of OS in this phenomenon [186].

In a prospective cohort investigation involving 41,620 patients with a history of stroke or AMI who received a dose of 83–201 mg/day of ascorbate, it was found that after a follow-up of 7.9 years, vitamin C was associated with a statistically lower risk of IS [187].

Despite encouraging evidence, controversy persists. A large study found no benefit from the supplementation of 400 intenational units (IU) of vitamin E every other day and 500 mg daily of vitamin C in middle-aged and elderly men [188]. Similar results were obtained with a supplementation dose of 250 mg/day of ascorbate for 5 years [189]. In this context, it is valid to mention that the evidence suggests that the dose administered is a crucial factor to induce the cardioprotective effect, significant amounts being necessary [190,191]. For example, in a recent meta-analysis involving 44 studies with a total of 1324 patients supplemented with doses between 500 and 2000 mg/day of ascorbate, a positive and significant impact was shown in the supplementation group in relation to endothelial function, which was measured through forearm blood flow (20 trials), flow-mediated dilatation (19 trials) and pulse wave (5 trials) [192].

Another alternative of interest lies in multiple treatment strategies, considering the synergistic effect of certain antioxidant compounds, such as the aforementioned ability of ascorbate to regenerate α-tocopherol. A study in our setting revealed that the combination of n-3 polyunsaturated fatty acids with vitamin C and E supplementation positively impacted the prevention of atrial fibrillation after surgery, which was associated with increased antioxidant potential and the mitigation of both OS and inflammation [193].

#### 5.3.2. Vitamin E

Alpha-tocopherol, commonly known as vitamin E, is a lipid-soluble vitamin with antioxidant properties that has several potential benefits for the human health. Indeed, vitamin E encompasses a group of tocopherols and tocotrienols which have been identified as possible anti-inflammatory, neuroprotective, anticarcinogenic, antihypertensive, atherogenesis inhibitor, antiallergic, antidiabetic, and telomerase activity modulators, in addition to being preventive against cardiovascular diseases, among other positive effects. It is present in several plant sources, especially in nuts such as walnuts and vegetable oils. It can also be found in foods that are part of the regular diet, such as vegetables, fruits, eggs, seafood and cheese [194].

It is recognized that this vitamin plays an essential role in safeguarding lipids against peroxidation [195], which in turn prevents this crucial event in the development and progression of atherosclerosis [196,197]. Therefore, it has been postulated that alpha-tocopherol could attenuate the atherosclerotic process and reduce the risk of cardiovascular disease. The main action of this compound is based on its ability to inhibit NOX and lipid peroxidation, which justifies its contribution to the reversal of endothelial dysfunction [176].

Numerous observational studies have shown that an increase in vitamin E intake, either through diet or supplementation, is associated with a decreased risk of cardiovascular disease [198,199]. For example, in a meta-analysis focused on the comparative efficacy of vitamin supplements in preventing major cardiovascular diseases, vitamin E was found to be effective in reducing the mortality rate linked to cardiovascular disease [200]. Nevertheless, a meta-analysis demonstrated that high-dosage (> or = 400 IU/day) vitamin E supplements may increase all-cause mortality and should be avoided [93].

Recently, a systematic review has been carried out that provided evidence for the positive effects of tocotrienols in models of brain injury and myocardial IRI. Tocotrienols were found to lead to significant improvements in structural, functional, and biochemical aspects in these models. Additionally, a marked reduction in OS, inflammation and apoptosis was observed because of tocotrienol treatment. These findings hint that tocotrienols may have therapeutic potential to counteract the adverse effects of IRI in both the brain and myocardium [201].

Another systematic review and meta-analysis indicated that the co-administration of omega-3 and vitamin E achieved a significant decrease in serum triglyceride (TG) and LDL levels in overweight patients with metabolic disorders [202]. A global analysis conducted by Cheng et al. found an inverse correspondence between high dietary vitamin E intake and overall stroke risk, showing a non-linear association [203].

However, as with vitamin C, controversies persist because several studies present inconsistent or even null results [204,205]. Thus, there is not yet unanimous agreement on the effects of vitamin E as monotherapy in the prevention and treatment of stroke. Several possible reasons could explain those inconsistent results, such as the different doses administered, the specific form of vitamin E used, the likely impact of proteins that regulate vitamin E homeostasis, and possible interactions of vitamin E with drugs, among other factors. Furthermore, it should be considered that many of these studies used vitamin E as a monotherapy. Taking into account the fact that the joint effects of vitamins C and E have been shown to be synergistic as ascorbate has the ability to reduce the α-tocopheroxyl radical to α-tocopherol, thus recycling vitamin E, while the antioxidant capacity of vitamin C is amplified when amalgamated with vitamin E [206], a multitherapy strategy is therefore an interesting alternative to consider.

#### 5.3.3. Vitamin D

Vitamin D is a secosterol known to regulate calcium and phosphate metabolism. It can be obtained from food and endogenously produced in the skin through sun exposure. There are two main dietary sources of vitamin D: cholecalciferol or vitamin D3, which can be found in foods such as oily fish or egg yolks and vitamin ergosterol or vitamin D2, which can be found in fungi and yeast [207]. Once in the bloodstream, vitamin D is transformed in the liver to 25-hydroxyvitamin D3, which in turn is further converted to the active form 1,25-dihydroxyvitamin D3 in the kidneys, being thus able to bind to its nuclear receptor to exert its physiologic functions [208].

Regarding its effects on OS and IRI, in vitro studies show that vitamin D3 exerts cardioprotective effects against myocardial IRI by protecting mitochondrial structural and functional integrity and reducing mitophagy [209]. When applied to a mouse model, vitamin D3 treatment mitigated mitochondrial fission, apoptosis, mitophagy, and myocardial structural abnormalities [209].

With respect to stroke, in a study in rodents given 1,25-dihydroxyvitamin D3 for one week and then subjected to 2 h of middle cerebral artery occlusion followed by 24 h of reperfusion, 1,25-dihydroxyvitamin D3 supplementation significantly reduced neurological deficit scores and areas of cerebral infarction, and it increased surviving neurons. This protective mechanism occurred through activation of the antioxidant Nrf2/HO-1 pathway to restrain NLRP3-mediated pyroptosis [83]. Thus, while further evidence is needed, especially in the clinical setting, vitamin D would be a safe compound with the potential to counteract myocardial and cerebral IRI.

#### 5.3.4. Folic Acid

Folic acid, a type of B vitamin essential in the regular human diet, is strongly associated with neuroinflammation [210]. It is also key for the development and function of the nervous system [86] and has been shown to have an antioxidant and a neuroprotective role [211]. A recent study assessed its potential role on synaptic structure and function, using an oxygen-glucose deprivation and reperfusion cell model, as well as a brain ischemia–reperfusion model. In this model, it was found that folic acid can effectively improve cognitive impairment and reduce neuronal death after cerebral ischemia, probably through improving synaptic dysfunction, concomitant with upregulated known markers of synaptic plasticity and downregulated N-methyl-D-aspartate receptor (NMDAR) expressions. Therefore, it could be suggested that folic acid may be a potential treatment to improve ischemic injury-induced cognitive decline [86].

In addition, another study, using a model of in vivo rats, as well as an oxygen–glucose deprivation/reoxygenation astrocyte cell model, found that in the context of ischemia–reperfusion, folic acid deficiency may develop an inflammatory response via the IL-6/JAK-1/phospho-STAT3 (pSTAT3) pathway on astrocytes, potentially leading to secondary brain injury. Therefore, folic acid supplementation was suggested as a potential preventive and therapeutic strategy to reduce brain damage in IS [210].

### 5.4. Trace Elements

Several trace elements are involved in the modulation of the activity of antioxidant enzymes, thereby contributing to the abrogation of OS occurring upon tissue reperfusion [212].

#### 5.4.1. Selenium

It has been suggested that Se has a great potential to ameliorate cerebral IRI. This process includes the Se-induced upregulation of mitofusin-1 expression, thus alleviating OS and ferroptosis, and the mechanism operates through the promotion of mitochondrial fusion both in vivo and in vitro [213].

The ability of the selenium compounds to increase GPX expression and activity has been related to their effectiveness in protecting against neuronal damage caused by cerebral ischemia–reperfusion in a murine model. From these studies, it was suggested that pretreatment with Se compounds provides neuronal protection in vivo against ischemia–reperfusion damage. Furthermore, the use of ferroptosis inhibitors can be suggested for treatment, whereas selenium compounds can be used for the prevention of IS-induced neuronal damage and other brain damage where ferroptosis is involved [214]. In addition, in a rat model of middle cerebral artery occlusion performed in hyperglycemic conditions, which is known to cause BBB impairment, it was found that Se inhibited autophagy and significantly prevented this damage by regulating the PI3K/Akt/mTOR signaling pathway [215].

Also, Se can exert cytoprotection in myocardial ischemia–reperfusion due to its role in the modulation of antioxidant enzymes, particularly GPX and thioredoxin reductases. The latter are selenocysteine-dependent enzymes, as Se is a cofactor regulating their gene expression. Therefore, Se supplementation may be a safe pharmacological resource for increasing antioxidant protection in the presence of risk of ischemia followed by reperfusion [216].

#### 5.4.2. Copper

Based on the occurrence of copper-induced Fenton reaction, this trace element provides a link toward pathophysiological cascades leading to OS and inflammation, playing a pathogenic role in stroke and ischemia–reperfusion myocardial damages. However, on the other hand, clinical data have suggested that plasma copper was significantly associated with a higher risk of IS [217], which was consistent with a previous study reporting the occurrence of significantly lower serum copper levels in patients with acute hemorrhagic stroke than in healthy control ones [218]. Moreover, it was suggested that increased dietary copper intake was associated with a lower risk of stroke [219]. Indeed, these conflicting results have been recently interpreted based on a double-edged sword whereby copper ions can exert in cells, suggesting that different organs may have unique optimal copper ion appropriate therapeutic concentrations. Therefore, the design and performance of more clinical trials are still necessary to establish future directions for copper ions treatments [220]

#### 5.4.3. Zinc

Zinc is a micronutrient essential to numerous biochemical pathways in human cells. In addition, salts of zinc have been used due to their protective effects against gastric, renal, hepatic, muscle, myocardial, or neuronal ischemic injury. With regard to target OS, Zn supplementation increased antioxidant parameters such as SOD, CAT, GPX, and GSH among others as well as Nrf2 expression and decreased mPTP opening, MDA and miRs-(122 and 34a), apoptotic factors, and histopathological changes [221].

The brain is an organ highly enriched in Zn content, the latter acting as a critical mediator of neuronal death during ischemia. An important mechanism of neuronal cell death in ischemic penumbra may be provided through the activation of endoplasmic reticulum stress by excessive Zn [222]. Studies of brain cerebral IRI by zinc accumulation in a murine model have reported that excessive zinc causes specific neuronal apoptosis induced by inflammation [223]. On the other hand, it has been suggested that Zn homeostasis plays a role in myocardial IRI, establishing an association between Zn deficiency and the development of cardiovascular diseases, which is supported by numerous studies. On this line, supplementing zinc can protect against myocardial infarction and IRI [224]. The endoplasmic reticulum stress/calcium-calmodulin-stimulated protein kinase II (CaMKII)/STAT3 axis may be an endogenous protective mechanism, which increases the resistance of the heart to ischemia–reperfusion. Studies in cardiomyocytes based on Zn deficiency suggested that the endoplasmic reticulum stress/CaMKII/STAT3 axis may be an endogenous protective mechanism that decreases the vulnerability of the heart to IRI [225].

#### 5.4.4. Manganese

Manganese is needed for the activity of manganese superoxide dismutase (MnSOD) or SOD2, which is one of the main antioxidant enzymes that protects the heart against IRI. This enzyme is a major mitochondrial antioxidant that scavenges O2•−. During ischemia followed by reperfusion, numerous studies have found a loss of SOD2 activity within the heart. Is important to note that the toxicity levels of Mn causing manganese stress, evoking increased ROS production and OS, are not well-defined. It was hypothesized that the affected energy metabolism is the primal cause of manganese toxicity. Manganese stress depletes cellular iron, affecting energy metabolism. In addition, impairment in the biogenesis of electron transport chain complexes causes ROS production [226].

### 5.5. Other Antioxidants

#### 5.5.1. Ginsenosides

The general structure of ginsenosides is a hydrophobic tetracyclic steroid skeleton, which is connected with sugar molecules and responsible for the hydrophilicity of the molecule [227]. *Panax ginseng Meyer* is a traditional Chinese herbal medicine in China whose pharmacological activities, such as anti-inflammatory, anti-oxidative, platelet aggregation-inhibiting, and neuronal apoptosis-suppressing effects [228], are mainly attributed to saponins, known as ginsenosides; and to other bioactive monomers like fatty acids, polysaccharides, and mineral oils [229]. In the following paragraphs, a more detailed description of ginsenoside Rg1 (G-Rg1) and ginsenoside Rb1 (G-Rb1) is made, due to their potential use in preventing ischemia after reperfusion in the brain [87] and heart [89,90], respectively. G-Rg1 exerts neurotrophic and neuroprotective pharmacological effects on the central nervous system [228]. In 2015, a meta-analysis was conducted to assess the neuroprotectant effect of G-Rb1 on IS in animal models, measuring the infarct volume and neurological function. The researchers found a significant improvement in experimental IS after G-Rg1 treatment [230]. When used in the context of cerebral IRI, G-Rg1 could reduce infarct size [87] as well as diminish neurological deficits and brain edema [231]. Furthermore, it was reported that this anti-IRI effect involves the Nrf2/ARE pathway [88]. There are beneficial biological activities of G-Rb1 in the cardiovascular systems, via the abrogation of OS, inflammation, and apoptosis [229]. A meta-analysis conducted in 2017 found a significant effect of G-Rb1 for decreasing the MI size compared with the control group in conjunction to decreasing plasma lactate dehydrogenase (LDH) and creatine kinase (CK) activities [89]. When used in the context of myocardial IRI, G-Rb1 diminishes infarct size, tissue injury, and apoptosis. Some mechanisms described are the regulation of the balance of RhoA signaling pathway [90] and the inhibition of mitochondrial complex I by reducing NADH dehydrogenase activity [91]. Also, its protective effects were particularly assessed in diabetic rats, and a beneficial enhance of Akt phosphorylation in the circumstance of myocardial IRI was found, leading to a reduction in postischemic myocardial infarct size and myocardial apoptosis [232].

#### 5.5.2. Erythropoietin

Several other protective pathways mediated by EPO have been reported including SIRT1 and JAK2/STAT pathways. The EPO-mediated activation of SIRT1 resulted in deacetylation and enhanced peroxisome proliferator-activated receptor gamma co-activator 1 alpha transcriptional activity which, in turn, promoted mitochondrial function and biogenesis in cardiomyocytes [233]. In fact, EPO pretreatment was demonstrated to decrease myocardial IRI [234]. Also, EPO decreases the cerebral ischemic area and the number of apoptotic cells in the ischemic penumbra in a rat model. These effects may be achieved via the EPO-mediated protection of cells against apoptosis [235].

#### 5.5.3. Estrogen

Estrogen deficiency is an important factor leading to cardiovascular diseases. 16α-hydroxyestrone, an estrogen-active component, could prevent cell death in myocardial tissue by regulating autophagy through the AMPK/mTOR pathway [236]. Also, a combined estrogen and progesterone treatment after ischemia could protect against glutamate neurotoxicity through modulating glutamate transporter expression, which in consequence induces glutamate re-uptake from extracellular space and prevents neurotoxicity [237].

## 6. Bases of Multitherapy for Improving Clinical Outcome

Preclinical models of reperfusion injury have shown beneficial effects of the administration of antioxidants and, thus, have contributed to understanding the underlying mechanisms. However, this benefit still has not been translated into clinical practice [238].

The knowledge of the potential benefits of the administration of antioxidants for preventing IRI and the particular pathways involved in the therapeutic effect of each compound calls our attention in order to hypothesize that a combination of different antioxidants, with different mechanisms of action, could produce synergic effects; indeed, this has been reported in several in vitro and in vivo studies [239].

To our knowledge, the combination of antioxidants has been mostly studied for assessing its role on AMI. In this regard, the combination of vitamin E with Crocin is promising, as it has been studied on a model of isolated heart of rat, whereas the reduction in infarct size was more marked compared with the effect of the antioxidants administered separately. The combination of vitamin C and vitamin E has been successfully studied for improving outcomes in patients suffering AMI [240,241] and also when combining both of them with vitamin A [28]. Also, While the combination of vitamin C and desferrioxamine did not show any significant protection against myocardial IRI on pigs [242], a study on the isolated ventricular cardiomyocytes and cardiac fibroblasts of neonatal rats, that combine both of them with N-acetylcysteine, showed a protection of cardiac fibroblasts against cell death; also, a recovering of pro-wound healing function was reported [243]. Although it is not a combination of two antioxidants, vitamin C was also studied in conjunction with N6-cyclopentyladenosine for preventing brain IRI, in a model of mice, with promising results [244].

The physiopathology of IRI on AMI and IS is a multifactorial phenomenon with the contribution of diverse causes including OS, inflammation, and autonomic nervous system dysfunction, among others. Although the monotherapies might not be able to compensate for the damage caused by ischemia followed by reperfusion, such as that occurring in AMI and IS subjected to reperfusion, it is of interest that several partial beneficial effects have been achieved using monotherapies. However, these effects may not be sufficient to produce a strong and robust effect in clinical situations where many uncontrolled variables usually coexist [245]. Therefore, considering the collaborative and potentially synergistic numerous actions of the various natural antioxidants, it should be expected that a multitarget protective antioxidant effect could lead to an improvement in the effectiveness of the decreased vulnerability of tissue to an oxidative challenge [3,27,245]. Accordingly, successful attempts with this aim have been performed [3]. Moreover, it has been shown that the combination of astaxanthin, lutein and zeaxanthin compared with a control group and a vitamin E group offered substantial improvement in cardioprotection against IRI and better outcomes [246]. Based on these data, we hypothesize that the combination of several natural antioxidants in patients with AMI undergoing percutaneous coronary intervention as well as IS patients could lead to an improvement in the cytoprotection against IRI.

In addition, even though this review focuses on the therapeutical approaches for IRI in the context of AMI and IS, many studies have been conducted for evaluating a multitherapy based on antioxidants to prevent ischemia after reperfusion in other situations, for example, in the context of heart surgery, intra-abdominal aortic aneurysm surgery that could lead to lower torso IRI [247], and renal IRI [248], showing promising results. Studies assessing multitherapy for decreasing ischemia after reperfusion are summarized in Table 2.

## 7. Concluding Remarks and Future Perspectives

OS plays an important role in the production mechanisms of damage caused by ischemia followed by reperfusion in the heart and brain. Increased ROS production and/or decreased antioxidant potential could be involved in biomolecules damage affecting the proteins, lipids, and DNA of these organs. The protective role of reinforcing of the ADS has been successfully demonstrated in experimental models but not yet translated into clinical settings. Likely, the use of monotherapies in the complex clinical damage caused in the heart and brain by multifactorial mechanisms could account for the reported discrepancies. Unfortunately, in most clinical studies, monotherapies have been more frequently assessed rather than multitherapies. In the present study, we present experimental and clinical evidence pointing out the hypothesis that the use of a combination of natural antioxidants should better than monotherapies with individual molecules to improve the beneficial effects of the reinforcement of the ADS. These compounds offer an effect that diminishes the adverse events occurring with the use of synthetic compounds. In addition, because natural antioxidants exhibit various protection mechanisms, from a combination of several diverse antioxidant molecules, a synergistic effect could be expected. It is of interest to suggest that randomized, placebo-controlled, double-blind, clinical trials are needed to test this hypothesis.

## 8. Methods

### 8.1. Study Design

Narrative Review

### 8.2. Research Question

Do natural antioxidants serve as effective prophylactic therapy in reducing IRI for patients undergoing reperfusion therapy for AMI and IS?

### 8.3. PICOST Strategy

- P: Population. Patients undergoing reperfusion therapy due to AMI or IS (cardiac arrest also was considered).
- I: Intervention. Prophylactic antioxidant therapy utilizing natural antioxidants.
- C: Comparators. Standard therapy. Also, combined therapies of natural antioxidants are considered to enhance the individual effects of the molecules; thus, they are compared to monotherapies.
- O: Outcomes. Any clinically relevant outcome.
- S: Study Designs. Randomized controlled trials, non-randomized studies, systematic reviews, and other relevant experimental studies (given the nature of this review).
- T: Timeframe. Inclusion of studies from all years (with a focus on the studies published in the last 10 years).

### 8.4. Search Strategy

A search was carried out for each antioxidant to investigate its potential applicability in both myocardial and cerebral contexts. The searches were conducted in PUBMED, encompassing the use of MeSH terms as well as searches without MeSH terms. Examples:

- (“Oxidative Stress” [Mesh]) AND (“Myocardial Reperfusion Injury” [Mesh]) AND (“Polyphenols” [Mesh])
- (“resveratrol” [title]) AND (“reperfusion injury” [title/abstract]) AND (“cerebral” [title/abstract])

Furthermore, relevant narrative reviews on the topic were consulted to identify additional literature.

### 8.5. Quality Assessment

Given the narrative review approach, formal quality assessment was omitted to comprehensively present diverse evidence from various study designs.

## Figures and Tables

**Table 1:** Examples of natural antioxidants candidates for improving clinical outcomes in patients undergoing MIRI and/or cerebral IRI.

Antioxidant Family	Antioxidant	Formula	Prevention Proposed	Target
Polyphenols	Resveratrol	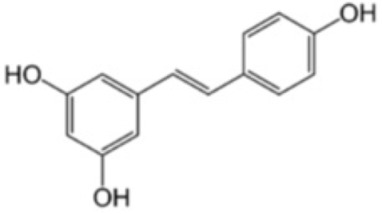	MIRI [60] and CIRI [61]	Ameliorate OS by increase in Nrf2 expression [61,62]. Also, decrease inflammation through TLR4/NF-κB signaling pathway [61,63].
	Quercetin	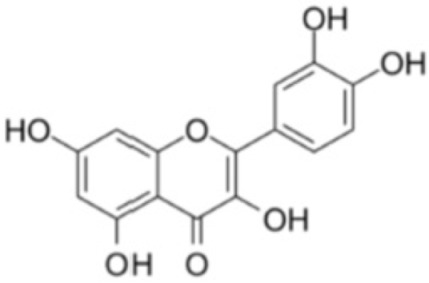	MIRI [64] and CIRI [65,66]	Scavenging and inhibition of ROS, and induction of Nrf2/HO-1 expression [65].
	Curcumin	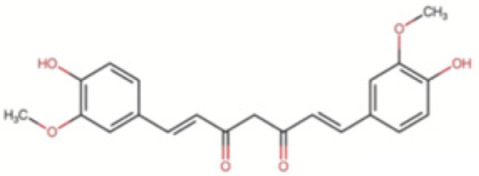	MIRI [67] and CIRI [68]	Decrease myocardial apoptosis by activating JAK2/STAT3 pathway, thus reducing OS-damage [67]; while neuroprotection could be due to inhibition of pyroptosis by suppressing the p38 MAPK pathway [69].
Carotenoids	Lycopene	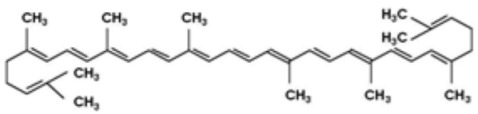	MIRI [33] and CIRI [70]	Inhibit mPTP opening via modulation of Bax and Bcl-2 [33,70].
	Crocin	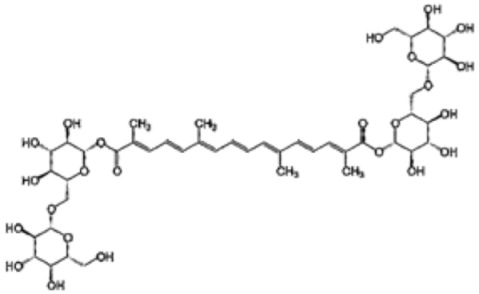	MIRI [71] and CIRI [58]	In the heart, there is a regulation of SIRT1/Nrf2 signaling and related endoplasmic reticulum stress [71], while in the brain, a reduction in HIF-1α and caspase-3 was seen [58].
	β-carotene	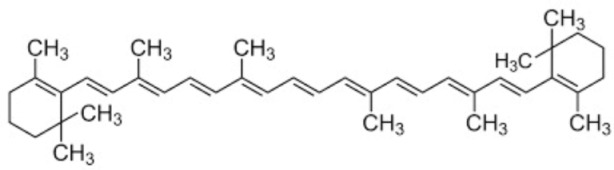	MIRI [72] and CIRI [73]	Inhibit NF-κB pathway [73].
	Astaxanthin	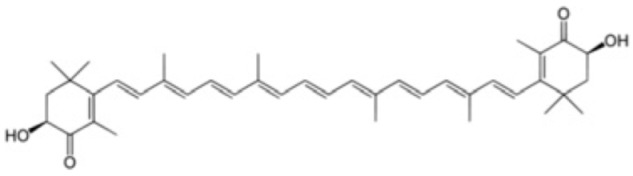	MIRI [74] and CIRI [75]	Activate Nrf2/HO-1 pathway, regulate the miR-138/HIF-1α axis [74]. Enhance the expressions of SOD1 and 2 [75]
	Lutein	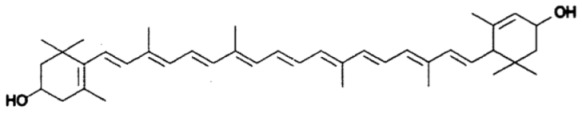	CIRI [76]	Decrease SOD, CAT and GTX activity [76].
Vitamins	All-trans retinoic acid (ATRA)	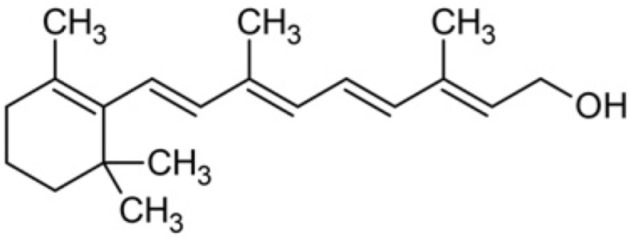	MIRI [77] and CIRI [78]	Downregulation of MAPK signaling [77,78].
	Vitamin C	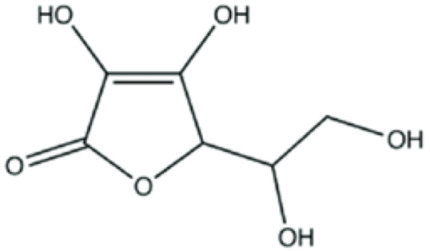	MIRI [79] and CIRI [80]	Decrease SOD activity [81] and PI3K-Akt signaling pathway [79].
	Vitamin D (α-tocopherol)	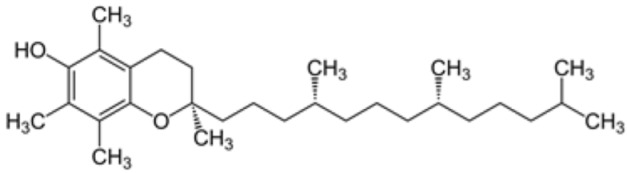	MIRI [82] and CIRI [83]	Reduce inflammation RhoA/ROCK/NF-ĸB pathway [82], activate Nrf2/HO-1 pathway, and suppress NLRP3-mediated pyroptotic pathway [83].
	Vitamin E	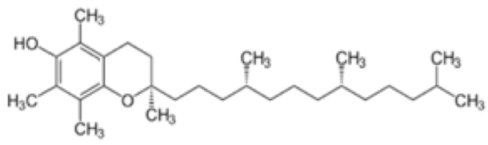	MIRI [84] and CIRI [85]	Downregulation of GPX (1, 5 and 6) and MPO [84].
	Folic acid	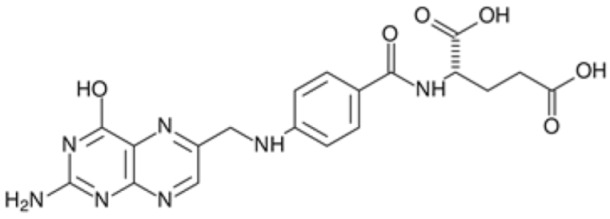	CIRI [86]	Inhibition of NMDAR [86].
Others	Ginsenoside Rg1	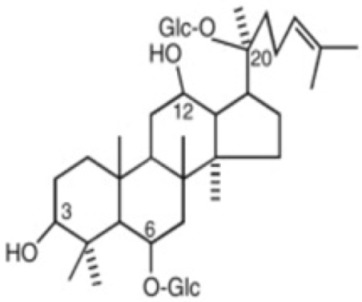	CIRI [87]	Activation of Nrf2/ARE pathway [88].
	Ginsenoside Rb1	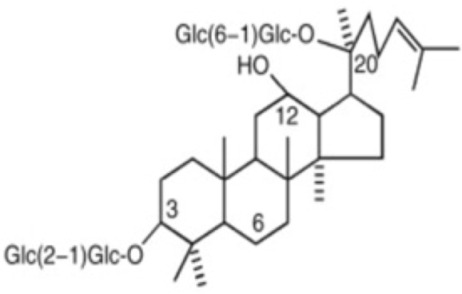	MIRI [89,90]	Reduce succinate-driven ROS production by inhibiting NADH dehydrogenase in mitochondrial complex I [91].

Akt: protein kinase B; ARE: antioxidant response elements; CIRI: cerebral ischemia–reperfusion injury; GPX: glutathione peroxidase; HO-1: heme oxygenase-1; MIRI: myocardial ischemia–reperfusion injury; MPO: myeloperoxidase; mPTP: mitochondrial permeability transition pore; NF-κB: nuclear factor kappa B; NMDAR: N-methyl-D-aspartate receptor; Nrf2: Nuclear factor erythroid 2-related factor 2; PI3K: phosphoinositide 3-kinase; ROS: reactive oxygen species; SIRT1: sirtuin 1; SOD: superoxide dismutase; TLR4: Toll-like receptor 4.

**Table 2:** Examples of studies assessing multitherapy for decreasing ischemia after reperfusion.

Drugs Used	Model	Results	Ref.
Myocardial ischemia–reperfusion injury			
Vitamin C Vitamin E Vitamin A	Randomized, double-blind, Clinical trial	Antioxidant therapy can reduce, in the first period after AMI, the OS, the inflammatory process, and the remodeling of the left ventricle and can affect the rate of a second heart attack in the short term. No comparison with each drug administered separately was performed.	[28]
Vitamin C Vitamin E	Randomized, double-blind, multicenter clinical trial	Composite of in-hospital cardiac mortality, non-fatal new myocardial infarction, VT/VF/asystole and shock/pulmonary edema occurred less frequently in patients treated with antioxidants.	[240]
Vitamin C Vitamin E	Randomized, double-blind, placebo-controlled clinical trial	Better preservation of cardiac function. No differences in CK-MB. Increased FRAP levels.	[241]
Vitamin E Crocin	Isolated heart of rats	The combination of drugs showed increased LVSP, improved contractility of heart and reduction in infarct size with superior effect compared with antioxidants administered separately.	[239]
Vitamin C Deferoxamine N-acetylcysteine	Isolated ventricular cardiomyocytes and cardiac fibroblasts of neonatal rats	Combination of all drugs protects cardiac fibroblasts from cell death and recovers pro-wound healing function damaged by simulated ischemia–reperfusion. Administration of each drug alone did not increase cell viability; however, treatments with the associations of vitamin C + deferoxamine and vitamin C + N-acetylcysteine also increased cell viability	[243]
Vitamin C Deferoxamine	Pigs	Does not provide significant protection against myocardial reperfusion injury.	[242]
Astaxanthin Lutein Zeaxanthin	Isolated heart of rats	The combination of drugs was compared with a control group and a vitamin E group. While the administration of vitamin E offered substantial cardioprotection to IRI, the mixture of astaxanthin, lutein and zeaxanthin protection had better outcomes. We observed reduced OS, infarct size and apoptosis, leading to an increase in myocardial function.	[246]
Cerebral ischemia– reperfusion injury			
Vitamin C N6-cyclopentyladenosine (CPA)	Mice	The group treated with both CPA and vitamin C showed a significant increase in density of normal cells in the CA1 region compared to the CPA or vitamin C alone groups.	[244]
Others			
Vitamin C Vitamin E EPA DHA	Randomized, double-blind, placebo-controlled clinical trial	Decrease in incidence of postoperative atrial fibrillation in patients over 60 years that underwent extracorporeal circulation.	[249]
Vitamin C Vitamin E EPA DHA	Randomized, double-blind, placebo-controlled clinical trial	Reduced the occurrence of postoperative atrial fibrillation on patients undergoing on-pump cardiac surgery.	[193]
Vitamin C Vitamin E EPA DHA	Randomized, double-blind, placebo-controlled clinical trial	No significant difference in the occurrence of postoperative atrial fibrillation in patients, aged 30–80 years, undergoing elective on-pump surgery (coronary artery bypass graft, valve surgery or mixed). However, lower inflammation-related parameters and MDA levels and a higher GSH/GSSG ratio was found in the supplemented group.	[250]
Vitamin C Vitamin E Hydrocortisone	Rats	In the context of renal ischemia–reperfusion injury, the simultaneous use of all drugs was more effective than using each drug alone.	[248]
Vitamin C Vitamin E Allopurinol N-acetylcysteine Mannitol	Randomized-controlled clinical trial. Not blinded	On lower torso ischemia and reperfusion after repair of intra-abdominal aortic aneurysm: Reduction in serum CK and ASAT.	[247]

AMI: acute myocardial infarction; ASAT: aspartate-aminotransferase; CK: creatine kinase; CK-MB: creatine kinase-myocardial band; CPA: N6-cyclopentyladenosine; FRAP: ferric-reducing ability of the plasma; GSH: glutathione; GSSG: glutathione disulfide; LVSP: left ventricular systolic pressure; MDA: malondialdehyde; OS: oxidative stress; VF: ventricular fibrillation; VT: ventricular tachycardia.

## Data Availability

No new data were created or analyzed in this study. Data sharing is not applicable to this article.

## References

[1] Abudurexiti A., Feng B., Nong Q., Zhang Y., Xu Z., He C., Huang H., Chen J., Gao H. (2023). Protective Effects of Chinese Herbal Monomers against Ischemia-Reperfusion Injury. Am. J. Transl. Res..

[2] Eren F., Yilmaz S. (2022). Neuroprotective Approach in Acute Ischemic Stroke: A Systematic Review of Clinical and Experimental Studies. Brain Circ..

[3] Rodrigo R., Retamal C., Schupper D., Vergara-Hernández D., Saha S., Profumo E., Buttari B., Saso L. (2022). Antioxidant Cardioprotection against Reperfusion Injury: Potential Therapeutic Roles of Resveratrol and Quercetin. Molecules.

[4] Herpich F., Rincon F. (2020). Management of Acute Ischemic Stroke. Crit. Care Med..

[5] Massalha S., Luria L., Kerner A., Roguin A., Abergel E., Hammerman H., Boulos M., Dragu R., Kapeliovich M.R., Beyar R. (2016). Heart Failure in Patients with Diabetes Undergoing Primary Percutaneous Coronary Intervention. Eur. Heart J. Acute Cardiovasc. Care.

[6] Wu M.-Y., Yiang G.-T., Liao W.-T., Tsai A.P.-Y., Cheng Y.-L., Cheng P.-W., Li C.-Y., Li C.-J. (2018). Current Mechanistic Concepts in Ischemia and Reperfusion Injury. Cell. Physiol. Biochem..

[7] Yellon D.M., Hausenloy D.J. (2007). Myocardial Reperfusion Injury. N. Engl. J. Med..

[8] Allen C.L., Bayraktutan U. (2009). Oxidative Stress and Its Role in the Pathogenesis of Ischaemic Stroke. Int. J. Stroke.

[9] Jennings R.B., Sommers H.M., Smyth G.A., Flack H.A., Linn H. (1960). Myocardial Necrosis Induced by Temporary Occlusion of a Coronary Artery in the Dog. Arch. Pathol..

[10] Ames A., Wright R.L., Kowada M., Thurston J.M., Majno G. (1968). Cerebral Ischemia. II. The No-Reflow Phenomenon. Am. J. Pathol..

[11] Shvedova M., Anfinogenova Y., Atochina-Vasserman E.N., Schepetkin I.A., Atochin D.N. (2018). C-Jun N-Terminal Kinases (JNKs) in Myocardial and Cerebral Ischemia/Reperfusion Injury. Front. Pharmacol..

[12] Kalogeris T., Baines C.P., Krenz M., Korthuis R.J. (2012). Cell Biology of Ischemia/Reperfusion Injury. International Review of Cell and Molecular Biology.

[13] Thengchaisri N., Hein T.W., Ren Y., Kuo L. (2015). Endothelin-1 Impairs Coronary Arteriolar Dilation: Role of P38 Kinase-Mediated Superoxide Production from NADPH Oxidase. J. Mol. Cell. Cardiol..

[14] Francis A., Baynosa R., Division of Plastic Surgery, Department of Surgery, University of Nevada School of Medicine, Las Vegas, USA (2017). Ischaemia-Reperfusion Injury and Hyperbaric Oxygen Pathways: A Review of Cellular Mechanisms. Diving Hyperb. Med..

[15] Zheng J., Chen P., Zhong J., Cheng Y., Chen H., He Y., Chen C. (2021). HIF-1α in Myocardial Ischemia-reperfusion Injury (Review). Mol. Med. Rep..

[16] Li J., Tao T., Xu J., Liu Z., Zou Z., Jin M. (2020). HIF-1α Attenuates Neuronal Apoptosis by Upregulating EPO Expression Following Cerebral Ischemia-reperfusion Injury in a Rat MCAO Model. Int. J. Mol. Med..

[17] Duan R., Pan H., Li D., Liao S., Han B. (2023). Ergothioneine Improves Myocardial Remodeling and Heart Function after Acute Myocardial Infarction via S-Glutathionylation through the NF-ĸB Dependent Wnt5a-SFlt-1 Pathway. Eur. J. Pharmacol..

[18] Zhou Y., Li K., Liu L., Li S. (2020). MicroRNA-132 Promotes Oxidative Stress-induced Pyroptosis by targeting Sirtuin 1 in Myocardial Ischaemia-reperfusion Injury. Int. J. Mol. Med..

[19] Kabłak-Ziembicka A., Badacz R., Przewłocki T. (2022). Clinical Application of Serum MicroRNAs in Atherosclerotic Coronary Artery Disease. J. Clin. Med..

[20] Zuo X., Lu J., Manaenko A., Qi X., Tang J., Mei Q., Xia Y., Hu Q. (2019). MicroRNA-132 Attenuates Cerebral Injury by Protecting Blood-Brain-Barrier in MCAO Mice. Exp. Neurol..

[21] Jin Z., Gan C., Luo G., Hu G., Yang X., Qian Z., Yao S. (2021). Notoginsenoside R1 Protects Hypoxia-Reoxygenation Deprivation-Induced Injury by Upregulation of MiR-132 in H9c2 Cells. Hum. Exp. Toxicol..

[22] Gong Y.-Y., Luo J.-Y., Wang L., Huang Y. (2018). MicroRNAs Regulating Reactive Oxygen Species in Cardiovascular Diseases. Antioxid. Redox Signal..

[23] Dhalla N.S., Shah A.K., Adameova A., Bartekova M. (2022). Role of Oxidative Stress in Cardiac Dysfunction and Subcellular Defects Due to Ischemia-Reperfusion Injury. Biomedicines.

[24] Rodrigo R., Prieto J.C., Castillo R. (2013). Cardioprotection against Ischaemia/Reperfusion by Vitamins C and E plus *n*−3 Fatty Acids: Molecular Mechanisms and Potential Clinical Applications. Clin. Sci..

[25] Xue H.-M., Sun W.-T., Chen H.-X., He G.-W., Yang Q. (2022). Targeting IRE1α-JNK-c-Jun/AP-1-SEH Signaling Pathway Improves Myocardial and Coronary Endothelial Function Following Global Myocardial Ischemia/Reperfusion. Int. J. Med. Sci..

[26] Mata A., Cadenas S. (2021). The Antioxidant Transcription Factor Nrf2 in Cardiac Ischemia–Reperfusion Injury. Int. J. Mol. Sci..

[27] Rodrigo R., Prieto J.C., Aguayo R., Ramos C., Puentes Á., Gajardo A., Panieri E., Rojas-Solé C., Lillo-Moya J., Saso L. (2021). Joint Cardioprotective Effect of Vitamin C and Other Antioxidants against Reperfusion Injury in Patients with Acute Myocardial Infarction Undergoing Percutaneous Coronary Intervention. Molecules.

[28] Gasparetto C., Malinverno A., Culacciati D., Gritt D., Prosperini P.G., Specchia G., Ricevuti G. (2005). Antioxidant Vitamins Reduce Oxidative Stress and Ventricular Remodeling in Patients with Acute Myocardial Infarction. Int. J. Immunopathol. Pharmacol..

[29] Hausenloy D.J., Yellon D.M. (2013). Myocardial Ischemia-Reperfusion Injury: A Neglected Therapeutic Target. J. Clin. Investig..

[30] Bagatini M.D., Martins C.C., Battisti V., Gasparetto D., da Rosa C.S., Spanevello R.M., Ahmed M., Schmatz R., Schetinger M.R.C., Morsch V.M. (2011). Oxidative Stress versus Antioxidant Defenses in Patients with Acute Myocardial Infarction. Heart Vessel..

[31] González-Montero J., Brito R., Gajardo A.I.J., Rodrigo R. (2018). Myocardial Reperfusion Injury and Oxidative Stress: Therapeutic Opportunities. World J. Cardiol..

[32] Cadenas S. (2018). ROS and Redox Signaling in Myocardial Ischemia-Reperfusion Injury and Cardioprotection. Free Radic. Biol. Med..

[33] Li X., Jia P., Huang Z., Liu S., Miao J., Guo Y., Wu N., Jia D. (2019). Lycopene Protects against Myocardial Ischemia-Reperfusion Injury by Inhibiting Mitochondrial Permeability Transition Pore Opening. Drug Des. Devel. Ther..

[34] Xie W., Santulli G., Reiken S.R., Yuan Q., Osborne B.W., Chen B.-X., Marks A.R. (2015). Mitochondrial Oxidative Stress Promotes Atrial Fibrillation. Sci. Rep..

[35] Wang C., Liu N., Luan R., Li Y., Wang D., Zou W., Xing Y., Tao L., Cao F., Wang H. (2013). Apelin Protects Sarcoplasmic Reticulum Function and Cardiac Performance in Ischaemia-Reperfusion by Attenuating Oxidation of Sarcoplasmic Reticulum Ca2+-ATPase and Ryanodine Receptor. Cardiovasc. Res..

[36] Santulli G., Lewis D., des Georges A., Marks A.R., Frank J. (2018). Ryanodine Receptor Structure and Function in Health and Disease. Subcellular Biochemistry.

[37] Zweier J.L., Flaherty J.T., Weisfeldt M.L. (1987). Direct Measurement of Free Radical Generation Following Reperfusion of Ischemic Myocardium. Proc. Natl. Acad. Sci. USA.

[38] Matsushima S., Sadoshima J. (2022). Yin and Yang of NADPH Oxidases in Myocardial Ischemia-Reperfusion. Antioxidants.

[39] Wang Q., Zuurbier C.J., Huhn R., Torregroza C., Hollmann M.W., Preckel B., van den Brom C.E., Weber N.C. (2023). Pharmacological Cardioprotection against Ischemia Reperfusion Injury—The Search for a Clinical Effective Therapy. Cells.

[40] Chouchani E.T., Pell V.R., Gaude E., Aksentijević D., Sundier S.Y., Robb E.L., Logan A., Nadtochiy S.M., Ord E.N.J., Smith A.C. (2014). Ischaemic Accumulation of Succinate Controls Reperfusion Injury through Mitochondrial ROS. Nature.

[41] Kabłak-Ziembicka A., Badacz R., Okarski M., Wawak M., Przewłocki T., Podolec J. (2023). Cardiac MicroRNAs: Diagnostic and Therapeutic Potential. Arch. Med. Sci..

[42] Dai B., Wang F., Nie X., Du H., Zhao Y., Yin Z., Li H., Fan J., Wen Z., Wang D.W. (2020). The Cell Type-Specific Functions of MiR-21 in Cardiovascular Diseases. Front. Genet..

[43] Lesizza P., Prosdocimo G., Martinelli V., Sinagra G., Zacchigna S., Giacca M. (2017). Single-Dose Intracardiac Injection of pro-Regenerative MicroRNAs Improves Cardiac Function after Myocardial Infarction. Circ. Res..

[44] Haghikia A., Missol-Kolka E., Tsikas D., Venturini L., Brundiers S., Castoldi M., Muckenthaler M.U., Eder M., Stapel B., Thum T. (2011). Signal Transducer and Activator of Transcription 3-Mediated Regulation of MiR-199a-5p Links Cardiomyocyte and Endothelial Cell Function in the Heart: A Key Role for Ubiquitin-Conjugating Enzymes. Eur. Heart J..

[45] Lu C., Jiang B., Xu J., Zhang X., Jiang N. (2023). Neferine Protected Cardiomyocytes against Hypoxia/Oxygenation Injury through SIRT1/Nrf2/HO-1 Signaling. J. Biochem. Mol. Toxicol..

[46] Ju J., Li X.-M., Zhao X.-M., Li F.-H., Wang S.-C., Wang K., Li R.-F., Zhou L.-Y., Liang L., Wang Y. (2023). Circular RNA FEACR Inhibits Ferroptosis and Alleviates Myocardial Ischemia/Reperfusion Injury by Interacting with NAMPT. J. Biomed. Sci..

[47] Shoaib M., Kim N., Choudhary R.C., Yin T., Shinozaki K., Becker L.B., Kim J. (2021). Increased Plasma Disequilibrium between Pro- and Anti-Oxidants during the Early Phase Resuscitation after Cardiac Arrest Is Associated with Increased Levels of Oxidative Stress End-Products. Mol. Med..

[48] Ishikawa K., Inoue Y., Sumi Y., Kondo Y., Okamoto K., Tanaka H. (2021). Novel Biomarkers of Oxidative Stress as Predictive Indicators of Neurological Outcome after Out-of-Hospital Cardiopulmonary Arrest. Am. J. Emerg. Med..

[49] Diao M.-Y., Zheng J., Shan Y., Xi S., Zhu Y., Hu W., Lin Z. (2020). Hypothermia Prevents Hippocampal Oxidative Stress and Apoptosis via the GSK-3β/Nrf2/HO-1 Signaling Pathway in a Rat Model of Cardiac Arrest-Induced Brain Damage. Neurol. Res..

[50] Hackenhaar F.S., Medeiros T.M., Heemann F.M., Behling C.S., Putti J.S., Mahl C.D., Verona C., da Silva A.C.A., Guerra M.C., Gonçalves C.A.S. (2017). Therapeutic Hypothermia Reduces Oxidative Damage and Alters Antioxidant Defenses after Cardiac Arrest. Oxid. Med. Cell. Longev..

[51] Orellana-Urzúa S., Claps G., Rodrigo R. (2021). Improvement of a Novel Proposal for Antioxidant Treatment against Brain Damage Occurring in Ischemic Stroke Patients. CNS Neurol. Disord. Drug Targets.

[52] Abramov A.Y., Scorziello A., Duchen M.R. (2007). Three Distinct Mechanisms Generate Oxygen Free Radicals in Neurons and Contribute to Cell Death during Anoxia and Reoxygenation. J. Neurosci..

[53] Zhang Q., Jia M., Wang Y., Wang Q., Wu J. (2022). Cell Death Mechanisms in Cerebral Ischemia-Reperfusion Injury. Neurochem. Res..

[54] Bernstein D.L., Zuluaga-Ramirez V., Gajghate S., Reichenbach N.L., Polyak B., Persidsky Y., Rom S. (2020). MiR-98 Reduces Endothelial Dysfunction by Protecting Blood-Brain Barrier (BBB) and Improves Neurological Outcomes in Mouse Ischemia/Reperfusion Stroke Model. J. Cereb. Blood Flow Metab..

[55] Yang Z., Huang C., Wen X., Liu W., Huang X., Li Y., Zang J., Weng Z., Lu D., Tsang C.K. (2022). Circular RNA Circ-FoxO3 Attenuates Blood-Brain Barrier Damage by Inducing Autophagy during Ischemia/Reperfusion. Mol. Ther..

[56] Basalay M.V., Davidson S.M., Yellon D.M. (2019). Neuroprotection in Rats Following Ischaemia-Reperfusion Injury by GLP-1 Analogues—Liraglutide and Semaglutide. Cardiovasc. Drugs Ther..

[57] Sarkaki A., Rashidi M., Ranjbaran M., Asareh Zadegan Dezfuli A., Shabaninejad Z., Behzad E., Adelipour M. (2021). Therapeutic Effects of Resveratrol on Ischemia–Reperfusion Injury in the Nervous System. Neurochem. Res..

[58] Oruc S., Gönül Y., Tunay K., Oruc O.A., Bozkurt M.F., Karavelioğlu E., Bağcıoğlu E., Coşkun K.S., Celik S. (2016). The Antioxidant and Antiapoptotic Effects of Crocin Pretreatment on Global Cerebral Ischemia Reperfusion Injury Induced by Four Vessels Occlusion in Rats. Life Sci..

[59] Shafi S., Ansari H.R., Bahitham W., Aouabdi S. (2019). The Impact of Natural Antioxidants on the Regenerative Potential of Vascular Cells. Front. Cardiovasc. Med..

[60] Mao Z.-J., Lin H., Hou J.-W., Zhou Q., Wang Q., Chen Y.-H. (2019). A Meta-Analysis of Resveratrol Protects against Myocardial Ischemia/Reperfusion Injury: Evidence from Small Animal Studies and Insight into Molecular Mechanisms. Oxid. Med. Cell. Longev..

[61] Xue R., Gao S., Zhang Y., Cui X., Mo W., Xu J., Yao M. (2022). A Meta-Analysis of Resveratrol Protects against Cerebral Ischemia/Reperfusion Injury: Evidence from Rats Studies and Insight into Molecular Mechanisms. Front. Pharmacol..

[62] Cheng L., Jin Z., Zhao R., Ren K., Deng C., Yu S. (2015). Resveratrol Attenuates Inflammation and Oxidative Stress Induced by Myocardial Ischemia-Reperfusion Injury: Role of Nrf2/ARE Pathway. Int. J. Clin. Exp. Med..

[63] Li J., Xie C., Zhuang J., Li H., Yao Y., Shao C., Wang H. (2014). Resveratrol Attenuates Inflammation in the Rat Heart Subjected to Ischemia-Reperfusion: Role of the TLR4/NF-ΚB Signaling Pathway. Mol. Med. Rep..

[64] Jin H.-B., Yang Y.-B., Song Y.-L., Zhang Y.-C., Li Y.-R. (2012). Protective Roles of Quercetin in Acute Myocardial Ischemia and Reperfusion Injury in Rats. Mol. Biol. Rep..

[65] Wang Y.-Y., Chang C.-Y., Lin S.-Y., Wang J.-D., Wu C.-C., Chen W.-Y., Kuan Y.-H., Liao S.-L., Wang W.-Y., Chen C.-J. (2020). Quercetin Protects against Cerebral Ischemia/Reperfusion and Oxygen Glucose Deprivation/Reoxygenation Neurotoxicity. J. Nutr. Biochem..

[66] Wang S., Chen Y., Xia C., Yang C., Chen J., Hai L., Wu Y., Yang Z. (2022). Synthesis and Evaluation of Glycosylated Quercetin to Enhance Neuroprotective Effects on Cerebral Ischemia-Reperfusion. Bioorg. Med. Chem..

[67] Zeng Y.-F., Guo Q.-H., Wei X.-Y., Chen S.-Y., Deng S., Liu J.-J., Yin N., Liu Y., Zeng W.-J. (2023). Cardioprotective Effect of Curcumin on Myocardial Ischemia/Reperfusion Injury: A Meta-Analysis of Preclinical Animal Studies. Front. Pharmacol..

[68] Yang X., Xu L., Zhao H., Xie T., Wang J., Wang L., Yang J. (2023). Curcumin Protects against Cerebral Ischemia-Reperfusion Injury in Rats by Attenuating Oxidative Stress and Inflammation: A Meta-Analysis and Mechanism Exploration. Nutr. Res..

[69] Huang L., Li X., Liu Y., Liang X., Ye H., Yang C., Hua L., Zhang X. (2021). Curcumin Alleviates Cerebral Ischemia-Reperfusion Injury by Inhibiting NLRP1-Dependent Neuronal Pyroptosis. Curr. Neurovasc. Res..

[70] Fujita K., Yoshimoto N., Kato T., Imada H., Matsumoto G., Inakuma T., Nagata Y., Miyachi E. (2013). Lycopene Inhibits Ischemia/Reperfusion-Induced Neuronal Apoptosis in Gerbil Hippocampal Tissue. Neurochem. Res..

[71] Wang X., Yuan B., Cheng B., Liu Y., Zhang B., Wang X., Lin X., Yang B., Gong G. (2019). Crocin Alleviates Myocardial Ischemia/Reperfusion-Induced Endoplasmic Reticulum Stress via Regulation of MiR-34a/Sirt1/Nrf2 Pathway. Shock.

[72] Csepanyi E., Gyongyosi A., Lekli I., Tosaki A., Bak I. (2022). Beta-Carotene Affects the Effects of Heme Oxygenase-1 in Isolated, Ischemic/Reperfused Rat Hearts: Potential Role of the Iron. Molecules.

[73] Althurwi H.N., Abdel-Rahman R.F., Soliman G.A., Ogaly H.A., Alkholifi F.K., Abd-Elsalam R.M., Alqasoumi S.I., Abdel-Kader M.S. (2022). Protective Effect of Beta-Carotene against Myeloperoxidase-Mediated Oxidative Stress and Inflammation in Rat Ischemic Brain Injury. Antioxidants.

[74] Zhang X., Xu M., Cai S., Chen B., Lin H., Liu Z. (2023). Effects of Astaxanthin on MicroRNA Expression in a Rat Cardiomyocyte Anoxia-Reoxygenation Model. Front. Pharmacol..

[75] Park J.H., Lee T.-K., Kim D.W., Ahn J.H., Lee C.-H., Kim J.-D., Shin M.C., Cho J.H., Lee J.-C., Won M.-H. (2022). Astaxanthin Confers a Significant Attenuation of Hippocampal Neuronal Loss Induced by Severe Ischemia-Reperfusion Injury in Gerbils by Reducing Oxidative Stress. Mar. Drugs.

[76] Sun Y.-X., Liu T., Dai X.-L., Zheng Q.-S., Hui B.-D., Jiang Z.-F. (2014). Treatment with Lutein Provides Neuroprotection in Mice Subjected to Transient Cerebral Ischemia. J. Asian Nat. Prod. Res..

[77] Zhu Z., Zhu J., Zhao X., Yang K., Lu L., Zhang F., Shen W., Zhang R. (2015). All-Trans Retinoic Acid Ameliorates Myocardial Ischemia/Reperfusion Injury by Reducing Cardiomyocyte Apoptosis. PLoS ONE.

[78] Li M., Tian X., An R., Yang M., Zhang Q., Xiang F., Liu H., Wang Y., Xu L., Dong Z. (2018). All-Trans Retinoic Acid Ameliorates the Early Experimental Cerebral Ischemia–Reperfusion Injury in Rats by Inhibiting the Loss of the Blood–Brain Barrier via the JNK/P38MAPK Signaling Pathway. Neurochem. Res..

[79] Hao J., Li W.-W., Du H., Zhao Z.-F., Liu F., Lu J.-C., Yang X.-C., Cui W. (2016). Role of Vitamin C in Cardioprotection of Ischemia/Reperfusion Injury by Activation of Mitochondrial K_ATP_ Channel. Chem. Pharm. Bull..

[80] Henry P.T., Chandy M.J. (1998). Effect of Ascorbic Acid on Infarct Size in Experimental Focal Cerebral Ischaemia and Reperfusion in a Primate Model. Acta Neurochir..

[81] Bhakuni P., Chandra M., Misra M.K. (2006). Effect of Ascorbic Acid Supplementation on Certain Oxidative Stress Parameters in the Post Reperfusion Patients of Myocardial Infarction. Mol. Cell. Biochem..

[82] Qian X., Zhu M., Qian W., Song J. (2019). Vitamin D Attenuates Myocardial Ischemia–Reperfusion Injury by Inhibiting Inflammation via Suppressing the RhoA/ROCK/NF-ĸB Pathway. Biotechnol. Appl. Biochem..

[83] Qiao J., Ma H., Chen M., Bai J. (2023). Vitamin D Alleviates Neuronal Injury in Cerebral Ischemia-Reperfusion via Enhancing the Nrf2/HO-1 Antioxidant Pathway to Counteract NLRP3-Mediated Pyroptosis. J. Neuropathol. Exp. Neurol..

[84] Wallert M., Ziegler M., Wang X., Maluenda A., Xu X., Yap M.L., Witt R., Giles C., Kluge S., Hortmann M. (2019). α-Tocopherol Preserves Cardiac Function by Reducing Oxidative Stress and Inflammation in Ischemia/Reperfusion Injury. Redox Biol..

[85] Salehi C., Seiiedy M., Soraya H., Fazli F., Ghasemnejad-Berenji M. (2021). Pretreatment with Bisoprolol and Vitamin E Alone or in Combination Provides Neuroprotection against Cerebral Ischemia/Reperfusion Injury in Rats. Naunyn. Schmiedebergs. Arch. Pharmacol..

[86] Liang X., Shi L., Wang M., Zhang L., Gong Z., Luo S., Wang X., Zhang Q., Zhang X. (2023). Folic Acid Ameliorates Synaptic Impairment Following Cerebral Ischemia/Reperfusion Injury via Inhibiting Excessive Activation of NMDA Receptors. J. Nutr. Biochem..

[87] Wang L., Zhao H., Zhai Z.-Z., Qu L.-X. (2018). Protective Effect and Mechanism of Ginsenoside Rg1 in Cerebral Ischaemia-Reperfusion Injury in Mice. Biomed. Pharmacother..

[88] Chu S.-F., Zhang Z., Zhou X., He W.-B., Chen C., Luo P., Liu D.-D., Ai Q.-D., Gong H.-F., Wang Z.-Z. (2019). Ginsenoside Rg1 Protects against Ischemic/Reperfusion-Induced Neuronal Injury through MiR-144/Nrf2/ARE Pathway. Acta Pharmacol. Sin..

[89] Zheng Q., Bao X.-Y., Zhu P.-C., Tong Q., Zheng G.-Q., Wang Y. (2017). Ginsenoside Rb1 for Myocardial Ischemia/Reperfusion Injury: Preclinical Evidence and Possible Mechanisms. Oxid. Med. Cell. Longev..

[90] Cui Y.-C., Pan C.-S., Yan L., Li L., Hu B.-H., Chang X., Liu Y.-Y., Fan J.-Y., Sun K., -Li Q. (2017). Ginsenoside Rb1 Protects against Ischemia/Reperfusion-Induced Myocardial Injury via Energy Metabolism Regulation Mediated by RhoA Signaling Pathway. Sci. Rep..

[91] Jiang L., Yin X., Chen Y.-H., Chen Y., Jiang W., Zheng H., Huang F.-Q., Liu B., Zhou W., Qi L.-W. (2021). Proteomic Analysis Reveals Ginsenoside Rb1 Attenuates Myocardial Ischemia/Reperfusion Injury through Inhibiting ROS Production from Mitochondrial Complex I. Theranostics.

[92] Siemann E.H., Creasy L.L. (1992). Concentration of the Phytoalexin Resveratrol in Wine. Am. J. Enol. Vitic..

[93] Bononi I., Tedeschi P., Mantovani V., Maietti A., Mazzoni E., Pancaldi C., Brandolini V., Tognon M. (2022). Antioxidant Activity of Resveratrol Diastereomeric Forms Assayed in Fluorescent-Engineered Human Keratinocytes. Antioxidants.

[94] Gambini J., López-Grueso R., Olaso-González G., Inglés M., Abdelazid K., El Alami M., Bonet-Costa V., Borrás C., Viña J. (2013). Resveratrol: Distribución, propiedades y perspectivas. Rev. Esp. Geriatr. Gerontol..

[95] Ro J.-H., Liu C.-C., Lin M.-C. (2020). Resveratrol Mitigates Cerebral Ischemic Injury by Altering Levels of Trace Elements, Toxic Metal, Lipid Peroxidation, and Antioxidant Activity. Biol. Trace Elem. Res..

[96] Li W., Ye A., Ao L., Zhou L., Yan Y., Hu Y., Fang W., Li Y. (2020). Protective Mechanism and Treatment of Neurogenesis in Cerebral Ischemia. Neurochem. Res..

[97] Tsai S.-K., Hung L.-M., Fu Y.-T., Cheng H., Nien M.-W., Liu H.-Y., Zhang F.B.-Y., Huang S.-S. (2007). Resveratrol Neuroprotective Effects during Focal Cerebral Ischemia Injury via Nitric Oxide Mechanism in Rats. J. Vasc. Surg..

[98] Li H., Yan Z., Zhu J., Yang J., He J. (2011). Neuroprotective Effects of Resveratrol on Ischemic Injury Mediated by Improving Brain Energy Metabolism and Alleviating Oxidative Stress in Rats. Neuropharmacology.

[99] Yan Y., Tong F., Chen J. (2019). Endogenous BMP-4/ROS/COX-2 Mediated IPC and Resveratrol Alleviated Brain Damage. Curr. Pharm. Des..

[100] Ren J., Fan C., Chen N., Huang J., Yang Q. (2011). Resveratrol Pretreatment Attenuates Cerebral Ischemic Injury by Upregulating Expression of Transcription Factor Nrf2 and HO-1 in Rats. Neurochem. Res..

[101] Gao Y., Fu R., Wang J., Yang X., Wen L., Feng J. (2018). Resveratrol Mitigates the Oxidative Stress Mediated by Hypoxic-Ischemic Brain Injury in Neonatal Rats via Nrf2/HO-1 Pathway. Pharm. Biol..

[102] Lei J., Tu X., Wang Y., Tu D., Shi S. (2019). Resveratrol Downregulates the TLR4 Signaling Pathway to Reduce Brain Damage in a Rat Model of Focal Cerebral Ischemia. Exp. Ther. Med..

[103] Khoury N., Xu J., Stegelmann S.D., Jackson C.W., Koronowski K.B., Dave K.R., Young J.I., Perez-Pinzon M.A. (2019). Resveratrol Preconditioning Induces Genomic and Metabolic Adaptations within the Long-Term Window of Cerebral Ischemic Tolerance Leading to Bioenergetic Efficiency. Mol. Neurobiol..

[104] Liu J., He J., Huang Y., Hu Z. (2021). Resveratrol Has an Overall Neuroprotective Role in Ischemic Stroke: A Meta-Analysis in Rodents. Front. Pharmacol..

[105] Banez M.J., Geluz M.I., Chandra A., Hamdan T., Biswas O.S., Bryan N.S., Von Schwarz E.R. (2020). A Systemic Review on the Antioxidant and Anti-Inflammatory Effects of Resveratrol, Curcumin, and Dietary Nitric Oxide Supplementation on Human Cardiovascular Health. Nutr. Res..

[106] Frankel E.N., Waterhouse A.L., Kinsella J.E. (1993). Inhibition of Human LDL Oxidation by Resveratrol. Lancet.

[107] Tadolini B., Juliano C., Piu L., Franconi F., Cabrini L. (2000). Resveratrol Inhibition of Lipid Peroxidation. Free Radic. Res..

[108] Wallerath T., Deckert G., Ternes T., Anderson H., Li H., Witte K., Förstermann U. (2002). Resveratrol, a Polyphenolic Phytoalexin Present in Red Wine, Enhances Expression and Activity of Endothelial Nitric Oxide Synthase. Circulation.

[109] Leikert J.F., Räthel T.R., Wohlfart P., Cheynier V., Vollmar A.M., Dirsch V.M. (2002). Red Wine Polyphenols Enhance Endothelial Nitric Oxide Synthase Expression and Subsequent Nitric Oxide Release from Endothelial Cells. Circulation.

[110] Gaggini M., Fenizia S., Vassalle C. (2023). Sphingolipid Levels and Signaling via Resveratrol and Antioxidant Actions in Cardiometabolic Risk and Disease. Antioxidants.

[111] Sun Z.-M., Guan P., Luo L.-F., Qin L.-Y., Wang N., Zhao Y.-S., Ji E.-S. (2020). Resveratrol Protects against CIH-Induced Myocardial Injury by Targeting Nrf2 and Blocking NLRP3 Inflammasome Activation. Life Sci..

[112] Kazemirad H., Kazerani H.R. (2020). Cardioprotective Effects of Resveratrol Following Myocardial Ischemia and Reperfusion. Mol. Biol. Rep..

[113] Feng L., Ren J., Li Y., Yang G., Kang L., Zhang S., Ma C., Li J., Liu J., Yang L. (2019). Resveratrol Protects against Isoproterenol Induced Myocardial Infarction in Rats through VEGF-B/AMPK/ENOS/NO Signalling Pathway. Free Radic. Res..

[114] Zhang Y.-M., Zhang Z.-Y., Wang R.-X. (2020). Protective Mechanisms of Quercetin Against Myocardial Ischemia Reperfusion Injury. Front. Physiol..

[115] Jiang W., Zhang H., Wu J., Zhai G., Li Z., Luan Y., Garg S. (2018). CuS@MOF-Based Well-Designed Quercetin Delivery System for Chemo–Photothermal Therapy. ACS Appl. Mater. Interfaces.

[116] Iskender H., Dokumacioglu E., Sen T.M., Ince I., Kanbay Y., Saral S. (2017). The Effect of Hesperidin and Quercetin on Oxidative Stress, NF-ΚB and SIRT1 Levels in a STZ-Induced Experimental Diabetes Model. Biomed. Pharmacother..

[117] Parasuraman S., Anand David A., Arulmoli R. (2016). Overviews of Biological Importance of Quercetin: A Bioactive Flavonoid. Pharmacogn. Rev..

[118] Luo M., Tian R., Yang Z., Peng Y.-Y., Lu N. (2019). Quercetin Suppressed NADPH Oxidase-Derived Oxidative Stress via Heme Oxygenase-1 Induction in Macrophages. Arch. Biochem. Biophys..

[119] Wan L.L., Xia J., Ye D., Liu J., Chen J., Wang G. (2009). Effects of Quercetin on Gene and Protein Expression of NOX and NOS after Myocardial Ischemia and Reperfusion in Rabbit. Cardiovasc. Ther..

[120] Duan J., Gao S., Tu S., Lenahan C., Shao A., Sheng J. (2021). Pathophysiology and Therapeutic Potential of NADPH Oxidases in Ischemic Stroke-Induced Oxidative Stress. Oxid. Med. Cell. Longev..

[121] Zhang C., Wang R., Zhang G., Gong D. (2018). Mechanistic Insights into the Inhibition of Quercetin on Xanthine Oxidase. Int. J. Biol. Macromol..

[122] Tian L., Cao W., Yue R., Yuan Y., Guo X., Qin D., Xing J., Wang X. (2019). Pretreatment with Tilianin Improves Mitochondrial Energy Metabolism and Oxidative Stress in Rats with Myocardial Ischemia/Reperfusion Injury via AMPK/SIRT1/PGC-1 Alpha Signaling Pathway. J. Pharmacol. Sci..

[123] Li J.-X., Tian R., Lu N. (2023). Quercetin Attenuates Vascular Endothelial Dysfunction in Atherosclerotic Mice by Inhibiting Myeloperoxidase and NADPH Oxidase Function. Chem. Res. Toxicol..

[124] Ulya T., Ardianto C., Anggreini P., Budiatin A.S., Setyawan D., Khotib J. (2021). Quercetin Promotes Behavioral Recovery and Biomolecular Changes of Melanocortin-4 Receptor in Mice with Ischemic Stroke. J. Basic Clin. Physiol. Pharmacol..

[125] Yang R., Shen Y.-J., Chen M., Zhao J.-Y., Chen S.-H., Zhang W., Song J.-K., Li L., Du G.-H. (2022). Quercetin Attenuates Ischemia Reperfusion Injury by Protecting the Blood-Brain Barrier through Sirt1 in MCAO Rats. J. Asian Nat. Prod. Res..

[126] Najda A., Klimek K., Balant S., Wrzesinska-Jedrusiak E., Piekarski W. (2019). Optimization of the Process of Polyphenol Extraction from Mentha Spicata with Various Solvents. Przem. Chem..

[127] Oliveira A.I., Pinho C., Sarmento B., Dias A.C.P. (2022). Quercetin-Biapigenin Nanoparticles Are Effective to Penetrate the Blood–Brain Barrier. Drug Deliv. Transl. Res..

[128] Riva A., Ronchi M., Petrangolini G., Bosisio S., Allegrini P. (2019). Improved Oral Absorption of Quercetin from Quercetin Phytosome^®^, a New Delivery System Based on Food Grade Lecithin. Eur. J. Drug Metab. Pharmacokinet..

[129] Barteková M., Šimončíková P., Fogarassyová M., Ivanová M., Okruhlicová Ľ., Tribulová N., Dovinová I., Barančík M. (2015). Quercetin Improves Postischemic Recovery of Heart Function in Doxorubicin-Treated Rats and Prevents Doxorubicin-Induced Matrix Metalloproteinase-2 Activation and Apoptosis Induction. Int. J. Mol. Sci..

[130] Chang X., Zhang T., Wang J., Liu Y., Yan P., Meng Q., Yin Y., Wang S. (2021). SIRT5-Related Desuccinylation Modification Contributes to Quercetin-Induced Protection against Heart Failure and High-Glucose-Prompted Cardiomyocytes Injured through Regulation of Mitochondrial Quality Surveillance. Oxid. Med. Cell. Longev..

[131] Dehghani F., Sezavar Seyedi Jandaghi S.H., Janani L., Sarebanhassanabadi M., Emamat H., Vafa M. (2021). Effects of Quercetin Supplementation on Inflammatory Factors and Quality of Life in Post-myocardial Infarction Patients: A Double Blind, Placebo-controlled, Randomized Clinical Trial. Phytother. Res..

[132] Sharifi-Rad J., Rayess Y.E., Rizk A.A., Sadaka C., Zgheib R., Zam W., Sestito S., Rapposelli S., Neffe-Skocińska K., Zielińska D. (2020). Turmeric and Its Major Compound Curcumin on Health: Bioactive Effects and Safety Profiles for Food, Pharmaceutical, Biotechnological and Medicinal Applications. Front. Pharmacol..

[133] Wang N.-P., Wang Z.-F., Tootle S., Philip T., Zhao Z.-Q. (2012). Curcumin Promotes Cardiac Repair and Ameliorates Cardiac Dysfunction Following Myocardial Infarction. Br. J. Pharmacol..

[134] Kotha R.R., Luthria D.L. (2019). Curcumin: Biological, Pharmaceutical, Nutraceutical, and Analytical Aspects. Molecules.

[135] Ran Y., Su W., Gao F., Ding Z., Yang S., Ye L., Chen X., Tian G., Xi J., Liu Z. (2021). Curcumin Ameliorates White Matter Injury after Ischemic Stroke by Inhibiting Microglia/Macrophage Pyroptosis through NF-ΚB Suppression and NLRP3 Inflammasome Inhibition. Oxid. Med. Cell. Longev..

[136] Wu S., Guo T., Qi W., Li Y., Gu J., Liu C., Sha Y., Yang B., Hu S., Zong X. (2021). Curcumin Ameliorates Ischemic Stroke Injury in Rats by Protecting the Integrity of the Blood-Brain Barrier. Exp. Ther. Med..

[137] Hou Y., Wang J., Feng J. (2019). The Neuroprotective Effects of Curcumin Are Associated with the Regulation of the Reciprocal Function between Autophagy and HIF-1α in Cerebral Ischemia-Reperfusion Injury. Drug Des. Dev. Ther..

[138] Huang L., Chen C., Zhang X., Li X., Chen Z., Yang C., Liang X., Zhu G., Xu Z. (2018). Neuroprotective Effect of Curcumin against Cerebral Ischemia-Reperfusion via Mediating Autophagy and Inflammation. J. Mol. Neurosci..

[139] Xie C.-J., Gu A.-P., Cai J., Wu Y., Chen R.-C. (2018). Curcumin Protects Neural Cells against Ischemic Injury in N2a Cells and Mouse Brain with Ischemic Stroke. Brain Behav..

[140] Tyagi N., Qipshidze N., Munjal C., Vacek J.C., Metreveli N., Givvimani S., Tyagi S.C. (2012). Tetrahydrocurcumin Ameliorates Homocysteinylated Cytochrome-c Mediated Autophagy in Hyperhomocysteinemia Mice after Cerebral Ischemia. J. Mol. Neurosci..

[141] Li T., Jin J., Pu F., Bai Y., Chen Y., Li Y., Wang X. (2023). Cardioprotective Effects of Curcumin against Myocardial I/R Injury: A Systematic Review and Meta-Analysis of Preclinical and Clinical Studies. Front. Pharmacol..

[142] Smirnova E., Moniruzzaman M., Chin S., Sureshbabu A., Karthikeyan A., Do K., Min T. (2023). A Review of the Role of Curcumin in Metal Induced Toxicity. Antioxidants.

[143] Gupta S.C., Prasad S., Kim J.H., Patchva S., Webb L.J., Priyadarsini I.K., Aggarwal B.B. (2011). Multitargeting by Curcumin as Revealed by Molecular Interaction Studies. Nat. Prod. Rep..

[144] Pereira D., Valentão P., Pereira J., Andrade P. (2009). Phenolics: From Chemistry to Biology. Molecules.

[145] Kumar N., Goel N. (2019). Phenolic Acids: Natural Versatile Molecules with Promising Therapeutic Applications. Biotechnol. Rep..

[146] Panda V., Laddha A., Nandave M., Srinath S. (2016). Dietary Phenolic Acids of *Macrotyloma Uniflorum* (Horse Gram) Protect the Rat Heart against Isoproterenol-Induced Myocardial Infarction. Phytother. Res..

[147] Liu G., Zhang B.-F., Hu Q., Liu X.-P., Chen J. (2020). Syringic Acid Mitigates Myocardial Ischemia Reperfusion Injury by Activating the PI3K/Akt/GSK-3β Signaling Pathway. Biochem. Biophys. Res. Commun..

[148] Draginic N., Milosavljevic I., Andjic M., Jeremic J., Nikolic M., Sretenovic J., Kocovic A., Srejovic I., Zivkovic V., Bolevich S. (2022). Short-Term Administration of Lemon Balm Extract Ameliorates Myocardial Ischemia/Reperfusion Injury: Focus on Oxidative Stress. Pharmaceuticals.

[149] Bai M., Liu B., Peng M., Jia J., Fang X., Miao M. (2019). Effect of Sargentodoxa Cuneata Total Phenolic Acids on Focal Cerebral Ischemia Reperfusion Injury Rats Model. Saudi J. Biol. Sci..

[150] Manochkumar J., Doss C.G.P., El-Seedi H.R., Efferth T., Ramamoorthy S. (2021). The Neuroprotective Potential of Carotenoids in Vitro and in Vivo. Phytomedicine.

[151] Khalid M., Saeed-ur-Rahman, Bilal M., Iqbal H.M.N., Huang D. (2019). Biosynthesis and Biomedical Perspectives of Carotenoids with Special Reference to Human Health-Related Applications. Biocatal. Agric. Biotechnol..

[152] Rao A.V., Rao L.G. (2007). Carotenoids and Human Health. Pharmacol. Res..

[153] Amengual J. (2019). Bioactive Properties of Carotenoids in Human Health. Nutrients.

[154] Hak A.E., Ma J., Powell C.B., Campos H., Gaziano J.M., Willett W.C., Stampfer M.J. (2004). Prospective Study of Plasma Carotenoids and Tocopherols in Relation to Risk of Ischemic Stroke. Stroke.

[155] Bahonar A., Saadatnia M., Khorvash F., Maracy M., Khosravi A. (2017). Carotenoids as Potential Antioxidant Agents in Stroke Prevention: A Systematic Review. Int. J. Prev. Med..

[156] Yang J., Zhang Y., Na X., Zhao A. (2022). Β-Carotene Supplementation and Risk of Cardiovascular Disease: A Systematic Review and Meta-Analysis of Randomized Controlled Trials. Nutrients.

[157] Kohlmeier L., Kark J.D., Gomez-Gracia E., Martin B.C., Steck S.E., Kardinaal A.F.M., Ringstad J., Thamm M., Masaev V., Riemersma R. (1997). Lycopene and Myocardial Infarction Risk in the EURAMIC Study. Am. J. Epidemiol..

[158] Bansal P., Gupta S.K., Ojha S.K., Nandave M., Mittal R., Kumari S., Arya D.S. (2006). Cardioprotective Effect of Lycopene in the Experimental Model of Myocardial Ischemia-Reperfusion Injury. Mol. Cell. Biochem..

[159] Upaganlawar A., Patel V., Balaraman R. (2012). Tomato Lycopene Attenuates Myocardial Infarction Induced by Isoproterenol: Electrocardiographic, Biochemical and Anti–Apoptotic Study. Asian Pac. J. Trop. Biomed..

[160] Hwa J.S., Jin Y.C., Lee Y.S., Ko Y.S., Kim Y.M., Shi L.Y., Kim H.J., Lee J.H., Ngoc T.M., Bae K.H. (2012). 2-Methoxycinnamaldehyde from Cinnamomum Cassia Reduces Rat Myocardial Ischemia and Reperfusion Injury in Vivo Due to HO-1 Induction. J. Ethnopharmacol..

[161] Tong C., Peng C., Wang L., Zhang L., Yang X., Xu P., Li J., Delplancke T., Zhang H., Qi H. (2016). Intravenous Administration of Lycopene, a Tomato Extract, Protects against Myocardial Ischemia-Reperfusion Injury. Nutrients.

[162] Efentakis P., Rizakou A., Christodoulou E., Chatzianastasiou A., López M.G., León R. (2017). Saffron (*Crocus Sativus*) Intake Provides Nutritional Preconditioning against Myocardial Ischemia-Reperfusion Injury in Wild Type and ApoE(−/−) Mice: Involvement of Nrf2 Activation. Nutr. Metab. Cardiovasc. Dis..

[163] Du Y., Ko K.M. (2006). Oleanolic Acid Protects against Myocardial Ischemia-Reperfusion Injury by Enhancing Mitochondrial Antioxidant Mechanism Mediated by Glutathione and α-Tocopherol in Rats. Planta Med..

[164] Ma B., Lu J., Kang T., Zhu M., Xiong K., Wang J. (2022). Astaxanthin Supplementation Mildly Reduced Oxidative Stress and Inflammation Biomarkers: A Systematic Review and Meta-Analysis of Randomized Controlled Trials. Nutr. Res..

[165] Cui G., Li L., Xu W., Wang M., Jiao D., Yao B., Xu K., Chen Y., Yang S., Long M. (2020). Astaxanthin Protects Ochratoxin A-Induced Oxidative Stress and Apoptosis in the Heart via the Nrf2 Pathway. Oxid. Med. Cell. Longev..

[166] Xue Y., Sun C., Hao Q., Cheng J. (2019). Astaxanthin Ameliorates Cardiomyocyte Apoptosis after Coronary Microembolization by Inhibiting Oxidative Stress via Nrf2/HO-1 Pathway in Rats. Naunyn. Schmiedebergs. Arch. Pharmacol..

[167] Zaafan M.A., Abdelhamid A.M. (2021). The Cardioprotective Effect of Astaxanthin against Isoprenaline-Induced Myocardial Injury in Rats: Involvement of TLR4/NF-ΚB Signaling Pathway. Eur. Rev. Med. Pharmacol. Sci..

[168] Gai Y.-S., Ren Y.-H., Gao Y., Liu H.-N. (2020). Astaxanthin Protecting Myocardial Cells from Hypoxia/Reoxygenation Injury by Regulating MiR-138/HIF-1α Axis. Eur. Rev. Med. Pharmacol. Sci..

[169] Cakir E., Cakir U., Tayman C., Turkmenoglu T.T., Gonel A., Turan I.O. (2020). Favorable Effects of Astaxanthin on Brain Damage Due to Ischemia-Reperfusion Injury. Comb. Chem. High Throughput Screen..

[170] Taheri F., Sattari E., Hormozi M., Ahmadvand H., Bigdeli M.R., Kordestani-Moghadam P., Anbari K., Milanizadeh S., Moghaddasi M. (2022). Dose-Dependent Effects of Astaxanthin on Ischemia/Reperfusion Induced Brain Injury in MCAO Model Rat. Neurochem. Res..

[171] Yang B.-B., Zou M., Zhao L., Zhang Y.-K. (2021). Astaxanthin Attenuates Acute Cerebral Infarction via Nrf-2/HO-1 Pathway in Rats. Curr. Res. Transl. Med..

[172] Levine M. (1999). Criteria and Recommendations for Vitamin C Intake. JAMA.

[173] Shen J., Griffiths P.T., Campbell S.J., Utinger B., Kalberer M., Paulson S.E. (2021). Ascorbate Oxidation by Iron, Copper and Reactive Oxygen Species: Review, Model Development, and Derivation of Key Rate Constants. Sci. Rep..

[174] Newaz M.A., Yousefipour Z., Nawal N.N.A. (2005). Modulation of Nitric Oxide Synthase Activity in Brain, Liver, and Blood Vessels of Spontaneously Hypertensive Rats by Ascorbic Acid: Protection from Free Radical Injury. Clin. Exp. Hypertens..

[175] Guney M., Oral B., Demirin H., Karahan N., Mungan T., Delibas N. (2007). Protective Effects of Vitamins C and E against Endometrial Damage and Oxidative Stress in Fluoride Intoxication. Clin. Exp. Pharmacol. Physiol..

[176] Ülker S., McKeown P.P., Bayraktutan U. (2003). Vitamins Reverse Endothelial Dysfunction through Regulation of ENOS and NAD(P)H Oxidase Activities. Hypertension.

[177] Wu F., Schuster D.P., Tyml K., Wilson J.X. (2007). Ascorbate Inhibits NADPH Oxidase Subunit P47phox Expression in Microvascular Endothelial Cells. Free Radic. Biol. Med..

[178] Gao F., Yao C.-L., Gao E., Mo Q.-Z., Yan W.-L., McLaughlin R., Lopez B.L., Christopher T.A., Ma X.L. (2002). Enhancement of Glutathione Cardioprotection by Ascorbic Acid in Myocardial Reperfusion Injury. J. Pharmacol. Exp. Ther..

[179] Packer J.E., Slater T.F., Willson R.L. (1979). Direct Observation of a Free Radical Interaction between Vitamin E and Vitamin C. Nature.

[180] Niki E., Noguchi N., Tsuchihashi H., Gotoh N. (1995). Interaction among Vitamin C, Vitamin E, and Beta-Carotene. Am. J. Clin. Nutr..

[181] May J.M., Qu Z.-C., Mendiratta S. (1998). Protection and Recycling of α-Tocopherol in Human Erythrocytes by Intracellular Ascorbic Acid. Arch. Biochem. Biophys..

[182] Chang C.-Y., Chen J.-Y., Wu M.-H., Hu M.-L. (2020). Therapeutic Treatment with Vitamin C Reduces Focal Cerebral Ischemia-Induced Brain Infarction in Rats by Attenuating Disruptions of Blood Brain Barrier and Cerebral Neuronal Apoptosis. Free Radic. Biol. Med..

[183] Cong G., Yan R., Sachdev U. (2020). Low Serum Vitamin C Correlates with an Increased Risk of Peripheral Arterial Disease in Current Smokers: Results from NHANES 2003–2004. Int. J. Cardiol. Hypertens..

[184] Langlois M., Duprez D., Delanghe J., De Buyzere M., Clement D.L. (2001). Serum Vitamin C Concentration Is Low in Peripheral Arterial Disease and Is Associated with Inflammation and Severity of Atherosclerosis. Circulation.

[185] Jayedi A., Rashidy-Pour A., Parohan M., Zargar M.S., Shab-Bidar S. (2019). Dietary and Circulating Vitamin C, Vitamin E, β-Carotene and Risk of Total Cardiovascular Mortality: A Systematic Review and Dose–Response Meta-Analysis of Prospective Observational Studies. Public Health Nutr..

[186] Basili S., Tanzilli G., Mangieri E., Raparelli V., Di Santo S., Pignatelli P., Violi F. (2010). Intravenous Ascorbic Acid Infusion Improves Myocardial Perfusion Grade during Elective Percutaneous Coronary Intervention. JACC Cardiovasc. Interv..

[187] Buijsse B., Jacobs D.R., Steffen L.M., Kromhout D., Gross M.D. (2015). Plasma Ascorbic Acid, A Priori Diet Quality Score, and Incident Hypertension: A Prospective Cohort Study. PLoS ONE.

[188] Sesso H.D. (2008). Vitamins E and C in the Prevention of Cardiovascular Disease in Men. JAMA.

[189] Hercberg S., Galan P., Preziosi P., Bertrais S., Mennen L., Malvy D., Roussel A.-M., Favier A., Briançon S. (2004). The SU.VI.MAX Study. Arch. Intern. Med..

[190] Jackson T.S., Xu A., Vita J.A., Keaney J.F. (1998). Ascorbate Prevents the Interaction of Superoxide and Nitric Oxide Only at Very High Physiological Concentrations. Circ. Res..

[191] Morelli M.B., Gambardella J., Castellanos V., Trimarco V., Santulli G. (2020). Vitamin C and Cardiovascular Disease: An Update. Antioxidants.

[192] Ran L., Zhao W., Tan X., Wang H., Mizuno K., Takagi K., Zhao Y., Bu H. (2020). Association between Serum Vitamin C and the Blood Pressure: A Systematic Review and Meta-Analysis of Observational Studies. Cardiovasc. Ther..

[193] Rodrigo R., Korantzopoulos P., Cereceda M., Asenjo R., Zamorano J., Villalabeitia E., Baeza C., Aguayo R., Castillo R., Carrasco R. (2013). A Randomized Controlled Trial to Prevent Post-Operative Atrial Fibrillation by Antioxidant Reinforcement. J. Am. Coll. Cardiol..

[194] Miyazawa T., Burdeos G.C., Itaya M., Nakagawa K., Miyazawa T. (2019). Vitamin E: Regulatory Redox Interactions. IUBMB Life.

[195] Clarke M.W., Burnett J.R., Croft K.D. (2008). Vitamin E in Human Health and Disease. Crit. Rev. Clin. Lab. Sci..

[196] Harrison D., Griendling K.K., Landmesser U., Hornig B., Drexler H. (2003). Role of Oxidative Stress in Atherosclerosis. Am. J. Cardiol..

[197] Navab M., Ananthramaiah G.M., Reddy S.T., Van Lenten B.J., Ansell B.J., Fonarow G.C., Vahabzadeh K., Hama S., Hough G., Kamranpour N. (2004). Thematic Review Series: The Pathogenesis of Atherosclerosis the Oxidation Hypothesis of Atherogenesis: The Role of Oxidized Phospholipids and HDL. J. Lipid Res..

[198] Rimm E.B., Stampfer M.J., Ascherio A., Giovannucci E., Colditz G.A., Willett W.C. (1993). Vitamin E Consumption and the Risk of Coronary Heart Disease in Men. N. Engl. J. Med..

[199] Kushi L.H., Folsom A.R., Prineas R.J., Mink P.J., Wu Y., Bostick R.M. (1996). Dietary Antioxidant Vitamins and Death from Coronary Heart Disease in Postmenopausal Women. N. Engl. J. Med..

[200] Han J., Zhao C., Cai J., Liang Y. (2020). Comparative Efficacy of Vitamin Supplements on Prevention of Major Cardiovascular Disease: Systematic Review with Network Meta-Analysis. Complement. Ther. Clin. Pract..

[201] Ramli F.F., Ali A., Ibrahim N. (2021). Protective Effects of Tocotrienols in Cerebral and Myocardial Ischemia-Reperfusion Injury: A Systematic Review. Appl. Sci..

[202] Asbaghi O., Choghakhori R., Abbasnezhad A. (2019). Effect of Omega-3 and Vitamin E Co-Supplementation on Serum Lipids Concentrations in Overweight Patients with Metabolic Disorders: A Systematic Review and Meta-Analysis of Randomized Controlled Trials. Diabetes Metab. Syndr..

[203] Cheng P., Wang L., Ning S., Liu Z., Lin H., Chen S., Zhu J. (2018). Vitamin E Intake and Risk of Stroke: A Meta-Analysis. Br. J. Nutr..

[204] Köpcke W. (2019). Vitamin E and Mortality: A Critical Perspective of the Conflicting Meta-Analysis Outcomes. Vitamin E in Human Health.

[205] Vučković B.A., van Rein N., Cannegieter S.C., Rosendaal F.R., Lijfering W.M. (2015). Vitamin Supplementation on the Risk of Venous Thrombosis: Results from the MEGA Case-Control Study. Am. J. Clin. Nutr..

[206] Metodiewa D., Kochman A., Karolczak S. (1997). Evidence for Antiradical and Antioxidant Properties of Four Biologically Active N,N-Diethylaminoethyl Ethers of Flavanone Oximes: A Comparison with Natural Polyphenolic Flavonoid (Rutin) Action. Biochem. Mol. Biol. Int..

[207] de la Guía-Galipienso F., Martínez-Ferran M., Vallecillo N., Lavie C.J., Sanchis-Gomar F., Pareja-Galeano H. (2021). Vitamin D and Cardiovascular Health. Clin. Nutr..

[208] Charoenngam N., Shirvani A., Holick M.F. (2019). Vitamin D for Skeletal and Non-Skeletal Health: What We Should Know. J. Clin. Orthop. Trauma.

[209] Lee T.-L., Lee M.-H., Chen Y.-C., Lee Y.-C., Lai T.-C., Lin H.Y.-H., Hsu L.-F., Sung H.-C., Lee C.-W., Chen Y.-L. (2020). Vitamin D Attenuates Ischemia/Reperfusion-Induced Cardiac Injury by Reducing Mitochondrial Fission and Mitophagy. Front. Pharmacol..

[210] Cheng M., Liang X., Shi L., Zhang Q., Zhang L., Gong Z., Luo S., Wang X., Zhang X. (2023). Folic Acid Deficiency Exacerbates the Inflammatory Response of Astrocytes after Ischemia-reperfusion by Enhancing the Interaction between IL-6 and JAK-1/PSTAT3. CNS Neurosci. Ther..

[211] Novochadlo M., Goldim M.P., Bonfante S., Joaquim L., Mathias K., Metzker K., Machado R.S., Lanzzarin E., Bernades G., Bagio E. (2021). Folic Acid Alleviates the Blood Brain Barrier Permeability and Oxidative Stress and Prevents Cognitive Decline in Sepsis-Surviving Rats. Microvasc. Res..

[212] Barandier C., Tanguy S., Pucheu S., Boucher F., Leiris J. (1999). Effect of Antioxidant Trace Elements on the Response of Cardiac Tissue to Oxidative Stressa. Ann. N. Y. Acad. Sci..

[213] Shi Y., Han L., Zhang X., Xie L., Pan P., Chen F. (2022). Selenium Alleviates Cerebral Ischemia/Reperfusion Injury by Regulating Oxidative Stress, Mitochondrial Fusion and Ferroptosis. Neurochem. Res..

[214] Tuo Q.-Z., Masaldan S., Southon A., Mawal C., Ayton S., Bush A.I., Lei P., Belaidi A.A. (2021). Characterization of Selenium Compounds for Anti-Ferroptotic Activity in Neuronal Cells and after Cerebral Ischemia–Reperfusion Injury. Neurotherapeutics.

[215] Yang B., Li Y., Ma Y., Zhang X., Yang L., Shen X., Zhang J., Jing L. (2021). Selenium Attenuates Ischemia/Reperfusion Injury-induced Damage to the Blood-brain Barrier in Hyperglycemia through PI3K/AKT/MTOR Pathway-mediated Autophagy Inhibition. Int. J. Mol. Med..

[216] Venardos K., Kaye D. (2007). Myocardial Ischemia-Reperfusion Injury, Antioxidant Enzyme Systems, and Selenium: A Review. Curr. Med. Chem..

[217] Xiao Y., Yuan Y., Liu Y., Yu Y., Jia N., Zhou L., Wang H., Huang S., Zhang Y., Yang H. (2019). Circulating Multiple Metals and Incident Stroke in Chinese Adults. Stroke.

[218] Karadas S., Sayın R., Aslan M., Gonullu H., Katı C., Dursun R., Duran L., Gonullu E., Demir H. (2014). Serum Levels of Trace Elements and Heavy Metals in Patients with Acute Hemorrhagic Stroke. J. Membr. Biol..

[219] Yang L., Chen X., Cheng H., Zhang L. (2022). Dietary Copper Intake and Risk of Stroke in Adults: A Case-Control Study Based on National Health and Nutrition Examination Survey 2013–2018. Nutrients.

[220] Chen X., Cai Q., Liang R., Zhang D., Liu X., Zhang M., Xiong Y., Xu M., Liu Q., Li P. (2023). Copper Homeostasis and Copper-Induced Cell Death in the Pathogenesis of Cardiovascular Disease and Therapeutic Strategies. Cell Death Dis..

[221] Akbari G. (2020). Role of Zinc Supplementation on Ischemia/Reperfusion Injury in Various Organs. Biol. Trace Elem. Res..

[222] Zhao Y., Ding M., Yang N., Huang Y., Sun C., Shi W. (2022). Zinc Accumulation Aggravates Cerebral Ischemia/Reperfusion Injury through Inducing Endoplasmic Reticulum Stress. Neurochem. Res..

[223] Li W., Yang X., Ding M., Shi W., Huang Y., An Q., Qi Z., Zhao Y. (2023). Zinc Accumulation Aggravates Cerebral Ischemia/Reperfusion Injury by Promoting Inflammation. Front. Cell. Neurosci..

[224] Choi S., Liu X., Pan Z. (2018). Zinc Deficiency and Cellular Oxidative Stress: Prognostic Implications in Cardiovascular Diseases. Acta Pharmacol. Sin..

[225] Zhao H., Liu D., Yan Q., Bian X., Yu J., Wang J., Cheng X., Xu Z. (2022). Endoplasmic Reticulum Stress/Ca2+-Calmodulin-Dependent Protein Kinase/Signal Transducer and Activator of Transcription 3 Pathway Plays a Role in the Regulation of Cellular Zinc Deficiency in Myocardial Ischemia/Reperfusion Injury. Front. Physiol..

[226] Kaur G., Kumar V., Arora A., Tomar A., Ashish, Sur R., Dutta D. (2017). Affected Energy Metabolism under Manganese Stress Governs Cellular Toxicity. Sci. Rep..

[227] Sun Y., Yang Y., Liu S., Yang S., Chen C., Lin M., Zeng Q., Long J., Yao J., Yi F. (2022). New Therapeutic Approaches to and Mechanisms of Ginsenoside Rg1 against Neurological Diseases. Cells.

[228] Xie W., Zhou P., Sun Y., Meng X., Dai Z., Sun G., Sun X. (2018). Protective Effects and Target Network Analysis of Ginsenoside Rg1 in Cerebral Ischemia and Reperfusion Injury: A Comprehensive Overview of Experimental Studies. Cells.

[229] Ramli F.F., Ali A., Ibrahim N. (2022). Molecular-Signaling Pathways of Ginsenosides Rb in Myocardial Ischemia-Reperfusion Injury: A Mini Review. Int. J. Med. Sci..

[230] Xie C.-L., Wang W.-W., Xue X.-D., Zhang S.-F., Gan J., Liu Z.-G. (2015). A Systematic Review and Meta-Analysis of Ginsenoside-Rg1 (G-Rg1) in Experimental Ischemic Stroke. Sci. Rep..

[231] Li Y., Guan Y., Wang Y., Yu C.-L., Zhai F.-G., Guan L.-X. (2017). Neuroprotective Effect of the Ginsenoside Rg1 on Cerebral Ischemic Injury in Vivo and in Vitro Is Mediated by PPAR*γ*-Regulated Antioxidative and Anti-Inflammatory Pathways. Evid. Based Complement. Alternat. Med..

[232] Wu Y., Xia Z.-Y., Dou J., Zhang L., Xu J.-J., Zhao B., Lei S., Liu H.-M. (2011). Protective Effect of Ginsenoside Rb1 against Myocardial Ischemia/Reperfusion Injury in Streptozotocin-Induced Diabetic Rats. Mol. Biol. Rep..

[233] Chunchai T., Apaijai N., Benjanuwattra J., Pintana H., Singhanat K., Arunsak B., Chattipakorn N., Chattipakorn S.C. (2022). Erythropoietin Administration Exerted Neuroprotective Effects against Cardiac Ischemia/Reperfusion Injury. Curr. Res. Pharmacol. Drug Discov..

[234] Rong R., Xijun X. (2015). Erythropoietin Pretreatment Suppresses Inflammation by Activating the PI3K/Akt Signaling Pathway in Myocardial Ischemia-Reperfusion Injury. Exp. Ther. Med..

[235] Ma J.-Y., Jiang C.-J., Wang Z.-J., Zhao Y.-J., Zhang Z.-Y., Tao J.-J. (2016). Erythropoietin Reduces Apoptosis of Brain Tissue Cells in Rats after Cerebral Ischemia/Reperfusion Injury: A Characteristic Analysis Using Magnetic Resonance Imaging. Neural Regen. Res..

[236] Yin Z.-Y., Fu T., He S.-M., Fu L., Li X.-Z., Xu L., Du L., Yang T.-T., Zhu X., Wang C. (2023). 16α-OHE1, a Novel Oestrogen Metabolite, Attenuates Dysfunction of Left Ventricle Contractility via Regulation of Autophagy after Myocardial Ischemia and Reperfusion. Int. J. Cardiol..

[237] Nematipour S., Vahidinia Z., Nejati M., Naderian H., Beyer C., Azami Tameh A. (2020). Estrogen and Progesterone Attenuate Glutamate Neurotoxicity via Regulation of EAAT3 and GLT-1 in a Rat Model of Ischemic Stroke. Iran. J. Basic Med. Sci..

[238] San-Martín-Martínez D., Serrano-Lemus D., Cornejo V., Gajardo A.I.J., Rodrigo R. (2022). Pharmacological Basis for Abrogating Myocardial Reperfusion Injury through a Multi-Target Combined Antioxidant Therapy. Clin. Pharmacokinet..

[239] Dianat M., Esmaeilizadeh M., Badavi M., Samarbafzadeh A., Naghizadeh B. (2014). Protective Effects of Crocin on Hemodynamic Parameters and Infarct Size in Comparison with Vitamin E after Ischemia Reperfusion in Isolated Rat Hearts. Planta Med..

[240] Jaxa-Chamiec T., Bednarz B., Drozdowska D., Gessek J., Gniot J., Janik K., Kawka-Urbanek T., Maciejewski P., Ogórek M., Szpajer M. (2005). Antioxidant Effects of Combined Vitamins C and E in Acute Myocardial Infarction. The Randomized, Double-Blind, Placebo Controlled, Multicenter Pilot Myocardial Infarction and VITamins (MIVIT) Trial. Kardiol. Pol..

[241] Valls N., Gormaz J.G., Aguayo R., González J., Brito R., Hasson D., Libuy M., Ramos C., Carrasco R., Prieto J.C. (2016). Amelioration of Persistent Left Ventricular Function Impairment through Increased Plasma Ascorbate Levels Following Myocardial Infarction. Redox Rep..

[242] Chatziathanasiou G.N., Nikas D.N., Katsouras C.S., Kazakos N.D., Bouba V., Vougiouklakis T., Naka K.K., Michalis L.K. (2012). Combined Intravenous Treatment with Ascorbic Acid and Desferrioxamine to Reduce Myocardial Reperfusion Injury in an Experimental Model Resembling the Clinical Setting of Primary PCI. Hell. J. Cardiol..

[243] Parra-Flores P., Riquelme J.A., Valenzuela-Bustamante P., Leiva-Navarrete S., Vivar R., Cayupi-Vivanco J., Castro E., Espinoza-Pérez C., Ruz-Cortés F., Pedrozo Z. (2019). The Association of Ascorbic Acid, Deferoxamine and N-Acetylcysteine Improves Cardiac Fibroblast Viability and Cellular Function Associated with Tissue Repair Damaged by Simulated Ischemia/Reperfusion. Antioxidants.

[244] Zamani M., Katebi M., Mehdizadeh M., Kafami L., Soleimani M. (2013). Combination Therapy with A1 Receptor Agonist and Vitamin C Improved Working Memory in a Mouse Model of Global Ischemia-Reperfusion. Basic Clin. Neurosci..

[245] Davidson S.M., Ferdinandy P., Andreadou I., Bøtker H.E., Heusch G., Ibáñez B., Ovize M., Schulz R., Yellon D.M., Hausenloy D.J. (2019). Multitarget Strategies to Reduce Myocardial Ischemia/Reperfusion Injury. J. Am. Coll. Cardiol..

[246] Adluri R.S., Thirunavukkarasu M., Zhan L., Maulik N., Svennevig K., Bagchi M., Maulik G. (2013). Cardioprotective Efficacy of a Novel Antioxidant Mix VitaePro against Ex Vivo Myocardial Ischemia–Reperfusion Injury. Cell Biochem. Biophys..

[247] Wijnen M.H.W.A., Roumen R.M.H., Vader H.L., Goris R.J.A. (2002). A Multiantioxidant Supplementation Reduces Damage from Ischaemia Reperfusion in Patients after Lower Torso Ischaemia. A Randomised Trial. Eur. J. Vasc. Endovasc. Surg..

[248] Azari O., Kheirandish R., Azizi S., Farajli Abbasi M., Ghahramani Gareh Chaman S., Bidi M. (2015). Protective Effects of Hydrocortisone, Vitamin C and E Alone or in Combination against Renal Ischemia-Reperfusion Injury in Rat. Iran. J. Pathol..

[249] Rodrigo R., Gutierrez R., Fernandez R., Guzman P. (2012). Ageing Improves the Antioxidant Response against Postoperative Atrial Fibrillation: A Randomized Controlled Trial. Interact. Cardiovasc. Thorac. Surg..

[250] Castillo R., Rodrigo R., Perez F., Cereceda M., Asenjo R., Zamorano J., Navarrete R., Villalabeitia E., Sanz J., Baeza C. (2011). Antioxidant Therapy Reduces Oxidative and Inflammatory Tissue Damage in Patients Subjected to Cardiac Surgery with Extracorporeal Circulation. Basic Clin. Pharmacol. Toxicol..

